# Supplementary material for: PARP Inhibition Increases the Response to Chemotherapy in Uveal Melanoma
Source: Cancers (Basel). 2019 May 29;11(6):751. doi: 10.3390/cancers11060751 (PMC6628115; doi:10.3390/cancers11060751)
Supplement: Supplementary file 1 [file cancers-11-00751-s001.pdf]

Figure S1: Affymetrix-based PARP family gene expression

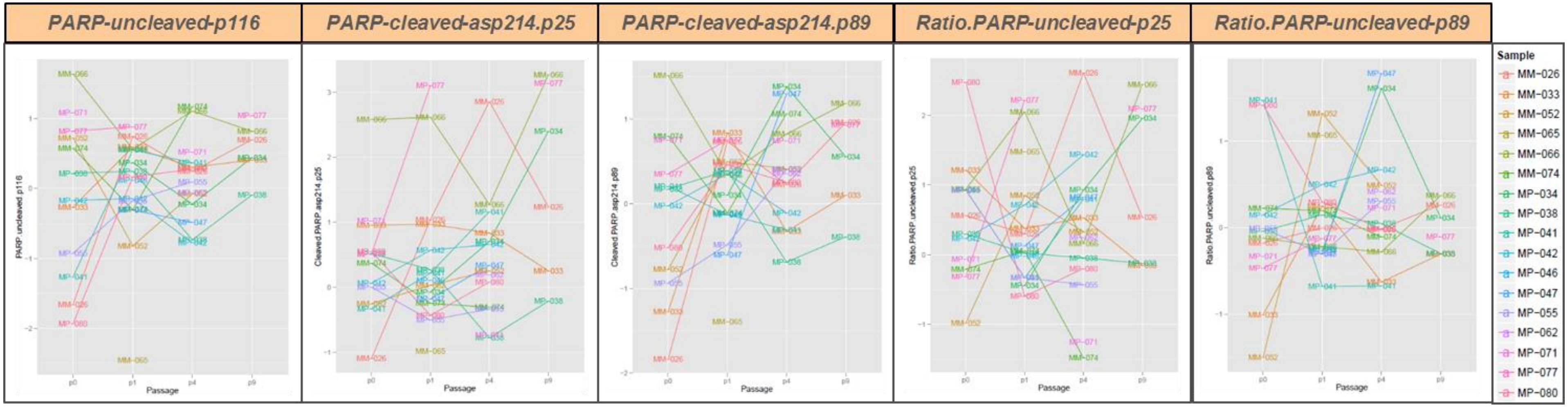

Figure S2: Affymetrix-based PARP family gene expression

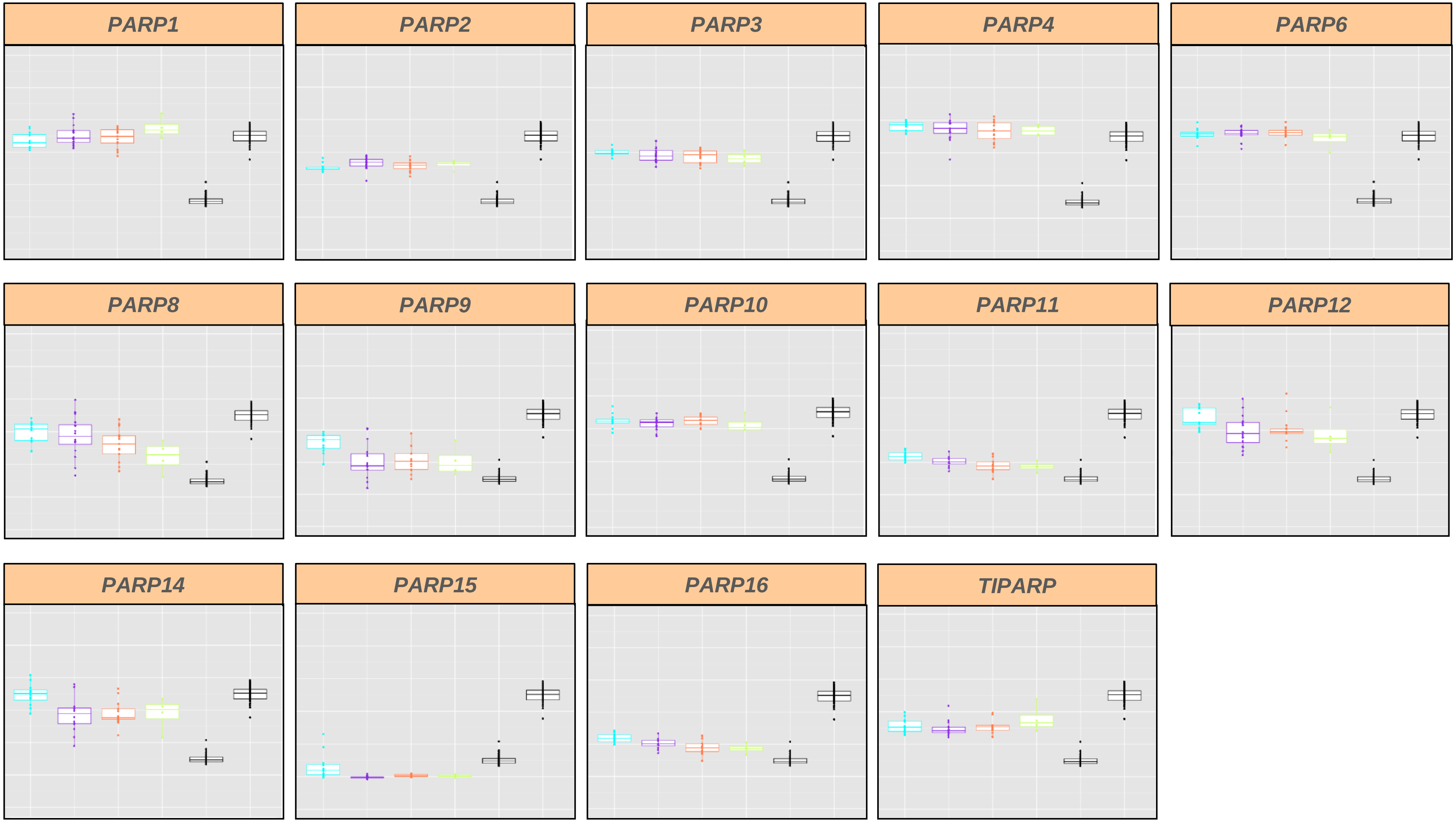

Figure S3: Combination of olaparib and fotemustine

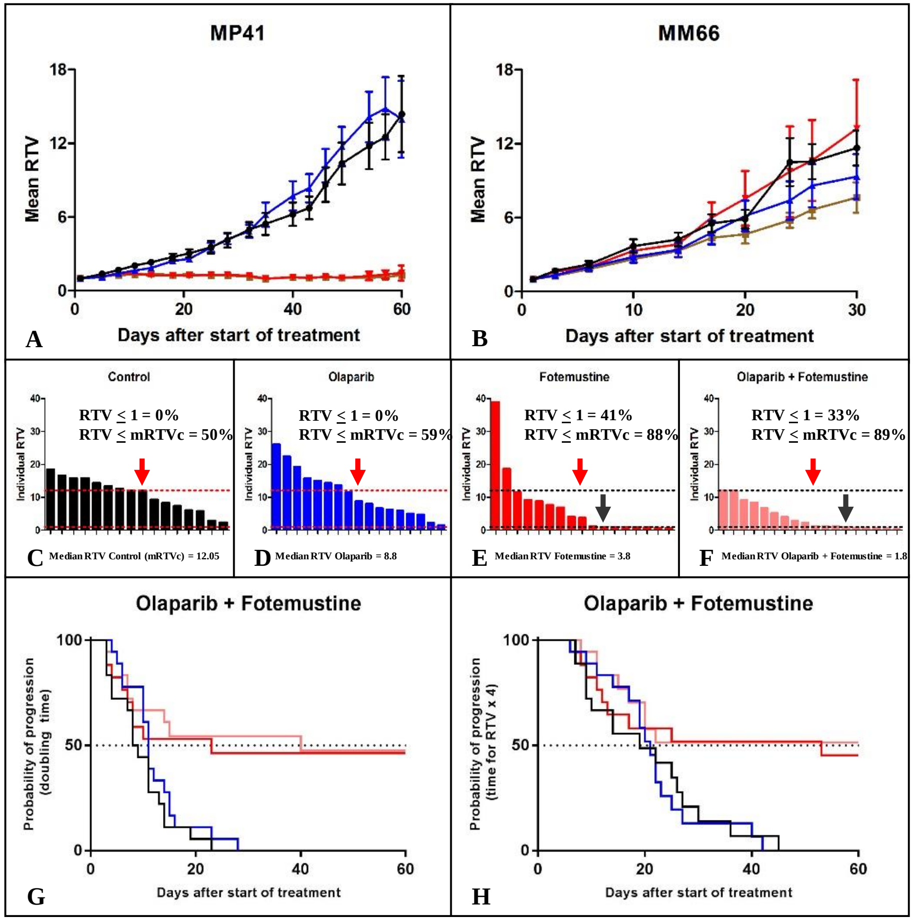

Figure S4: Combination of olaparib and everolimus

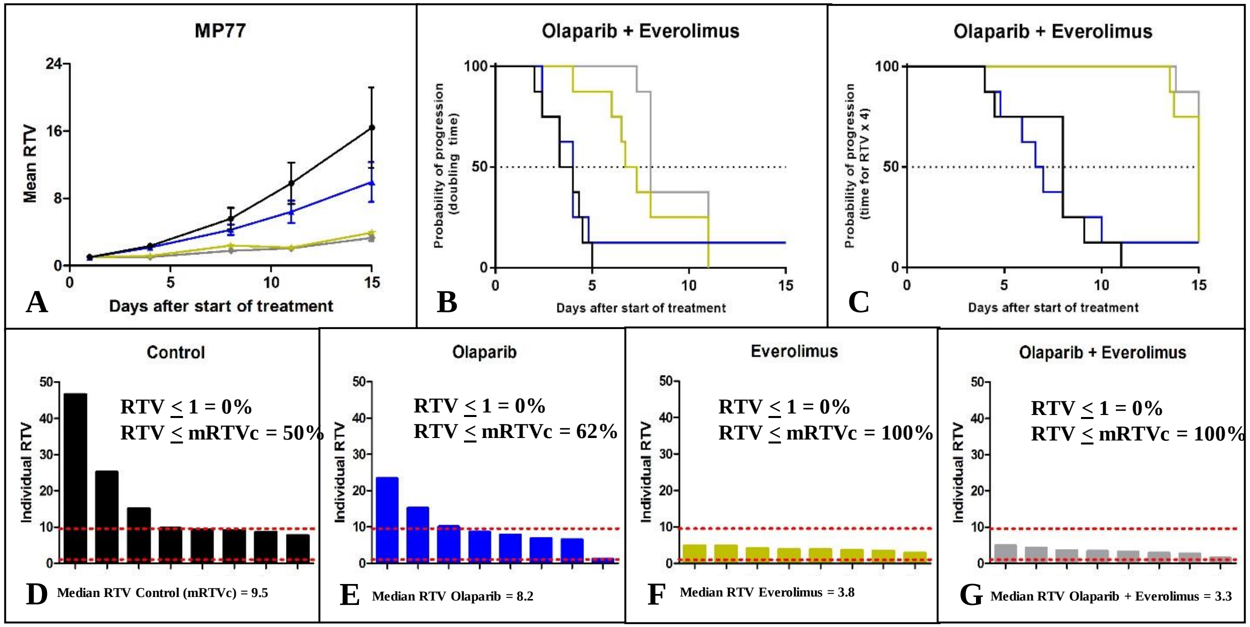

Figure S5: Combination of olaparib and AEB071

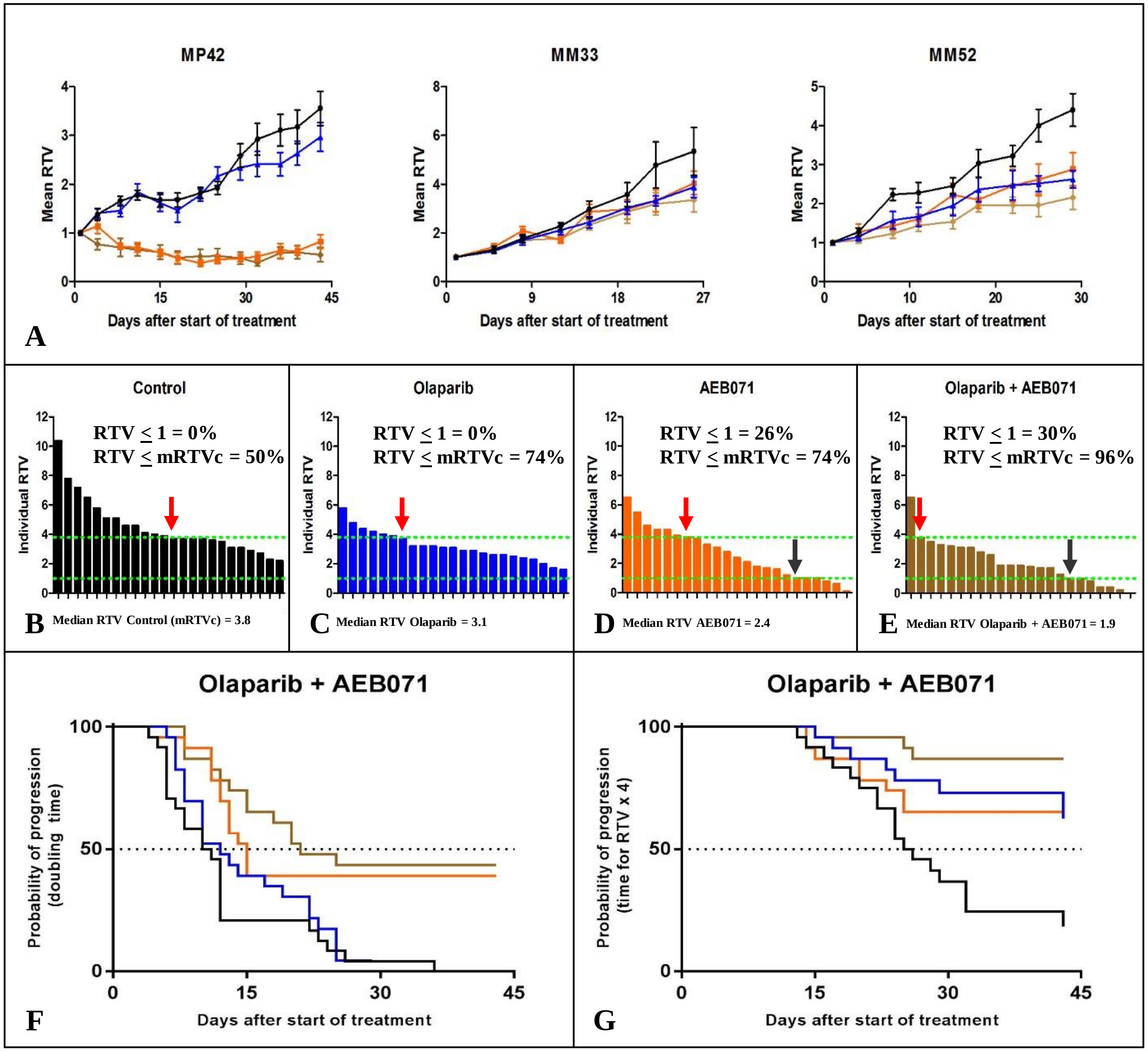

Figure S6: Combination of olaparib and CGM097

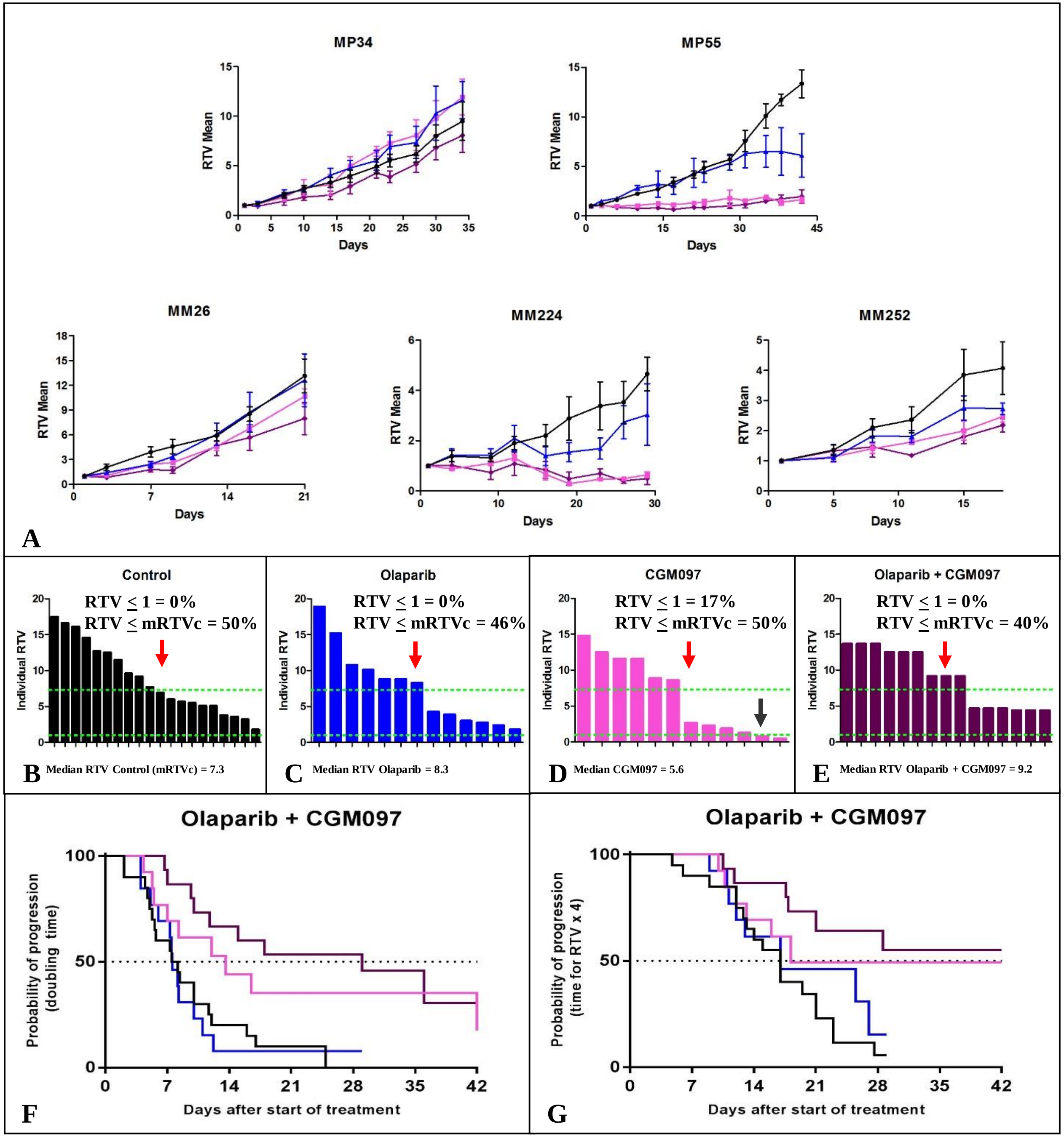

Figure S7: Combination of olaparib and AZD0156

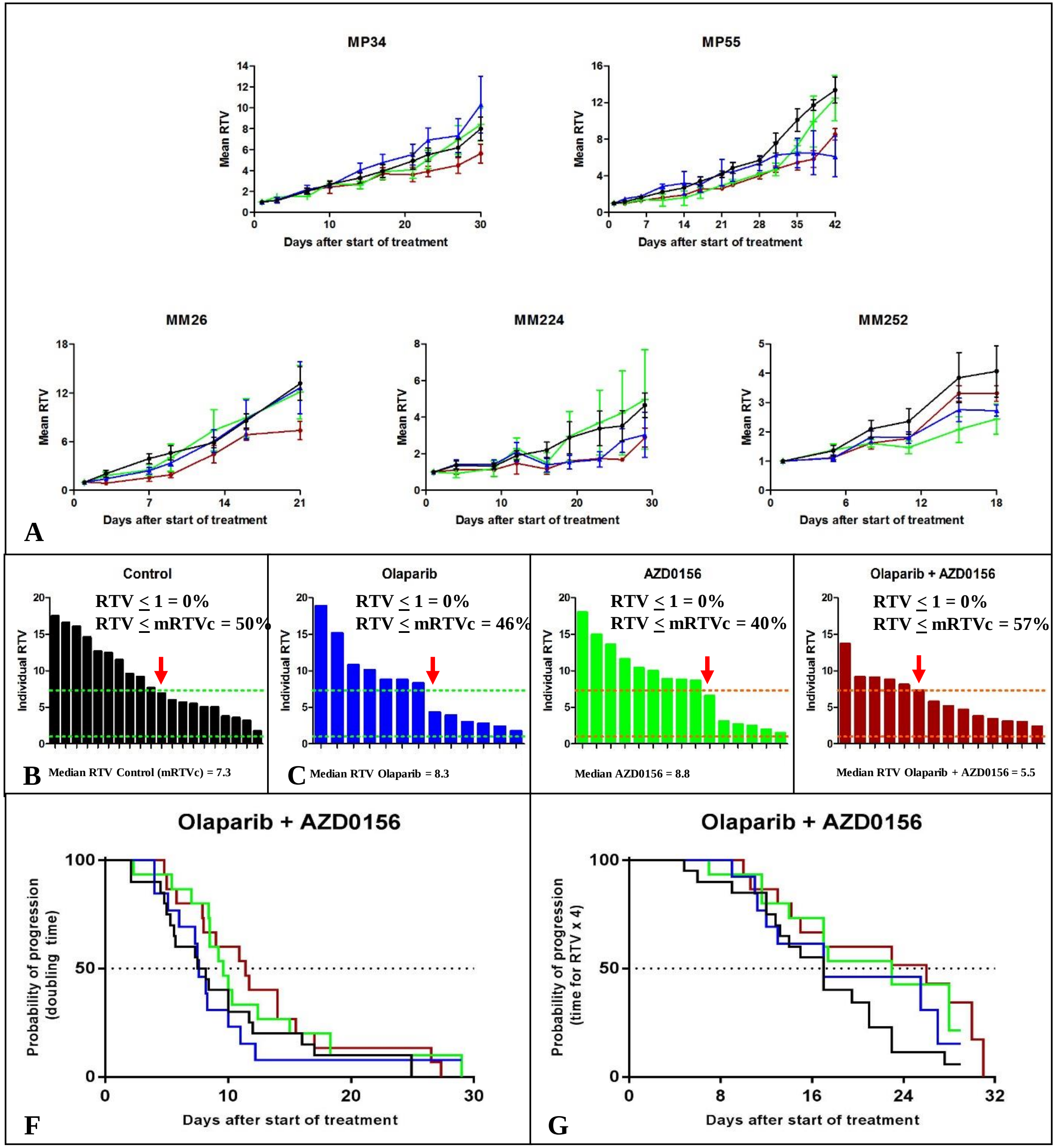

Figure S8: Combination of olaparib and AZD6738

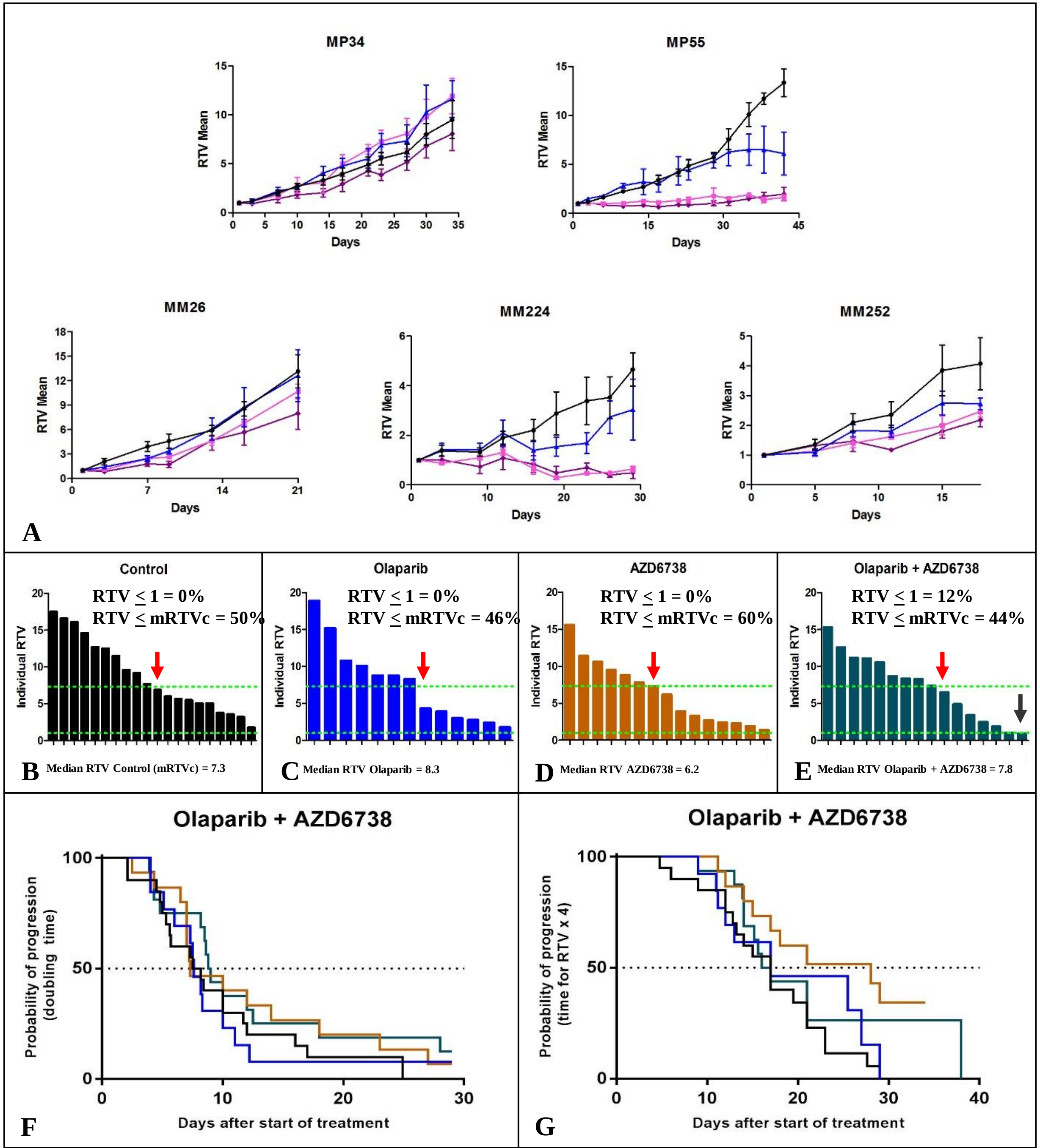

Figure S9: Significant protein expression between PDXs with additive efficacy (MP55-MP77-MM33) and PDX without additive efficacy (MM52) of the DTIC + olaparib combination ($p < 0.05$)

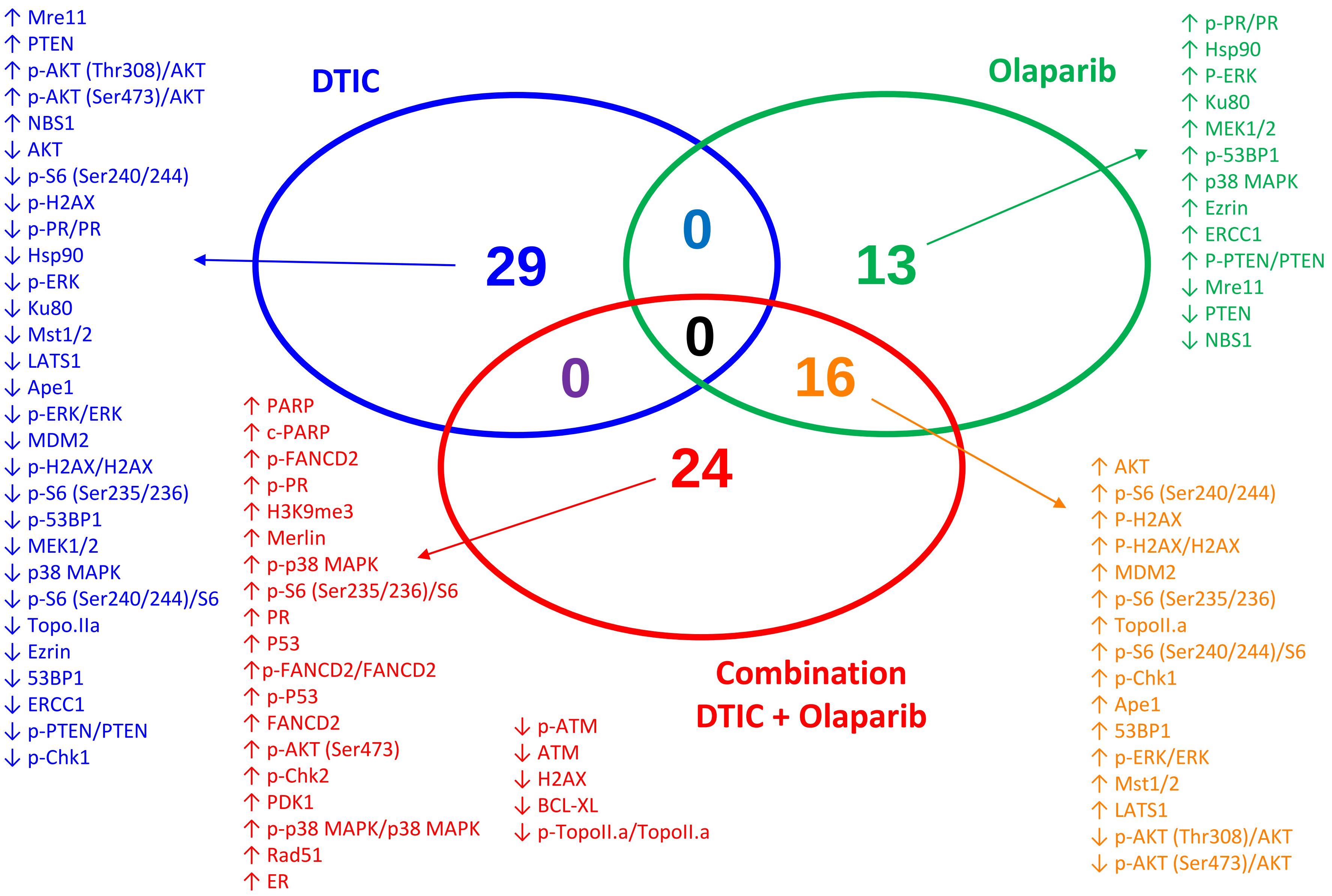

Figure S10: Significant protein expression between PDXs with additive efficacy (MP55-MP77-MM33) and PDX without additive efficacy (MM52) of the DTIC + olaparib combination

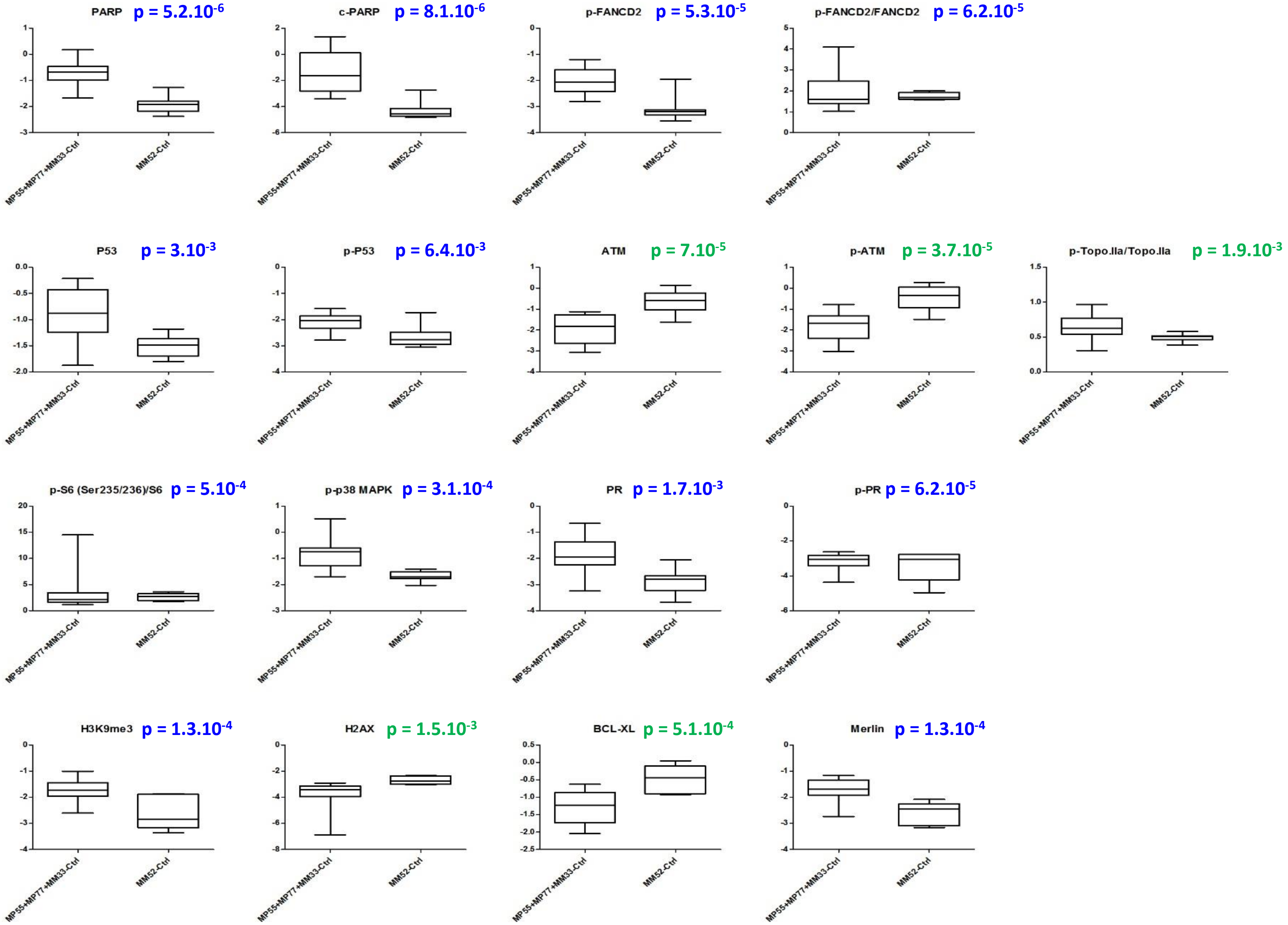

Figure S11: RPPA-based PARP and c-PARP protein expression

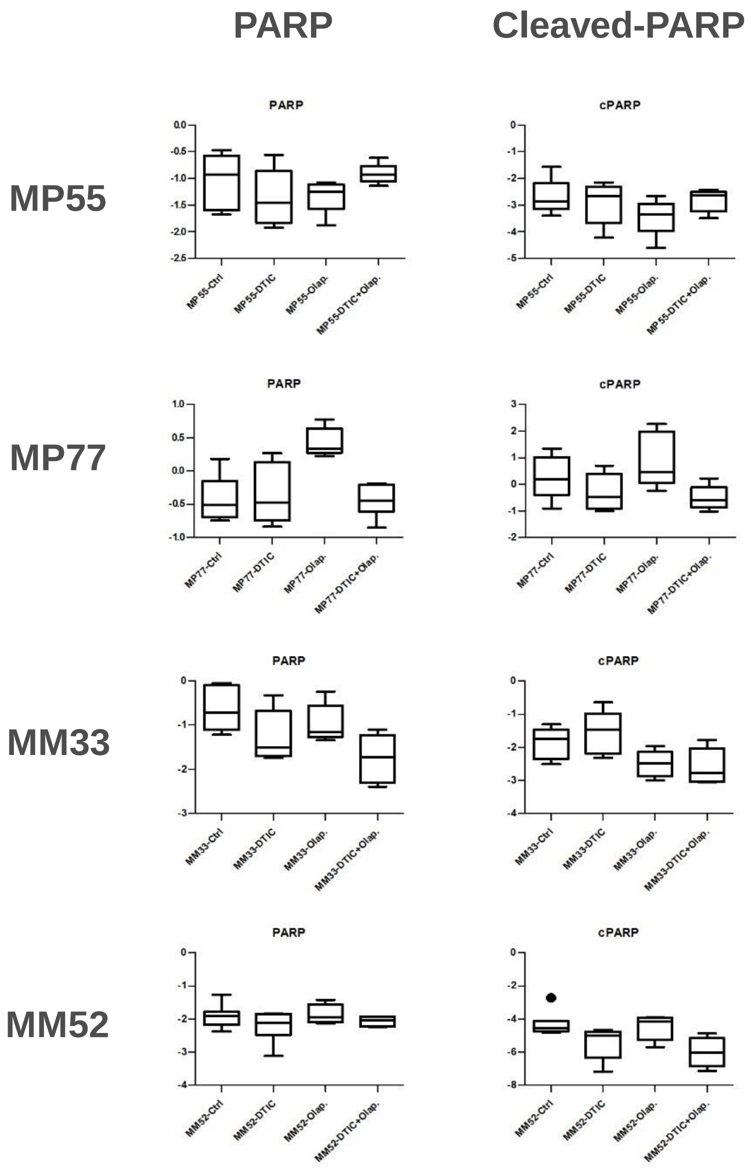

Figure S12: RPPA-based Apoptosis-related protein expression

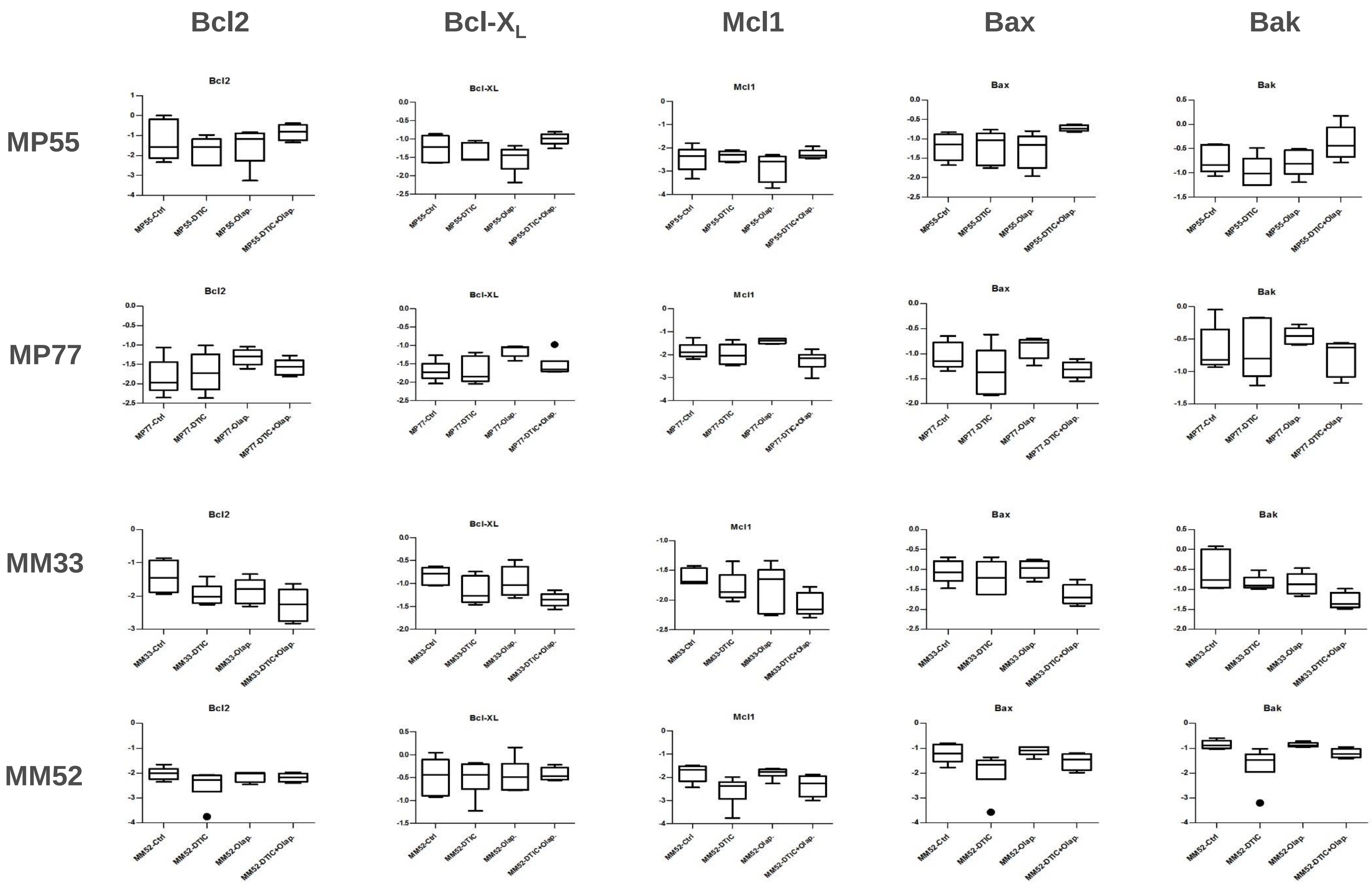

Figure S13: MP55 PDX

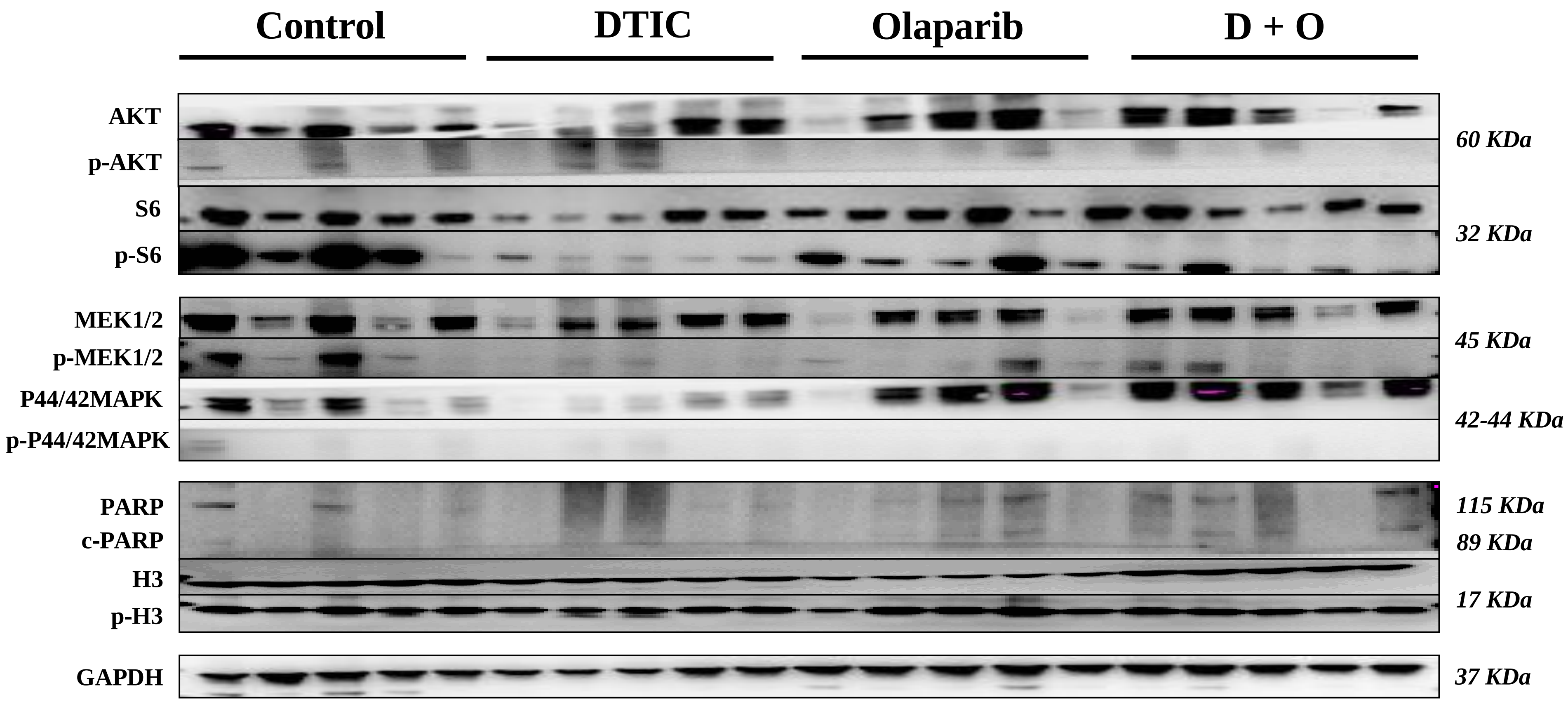

Figure S14: MP77 PDX

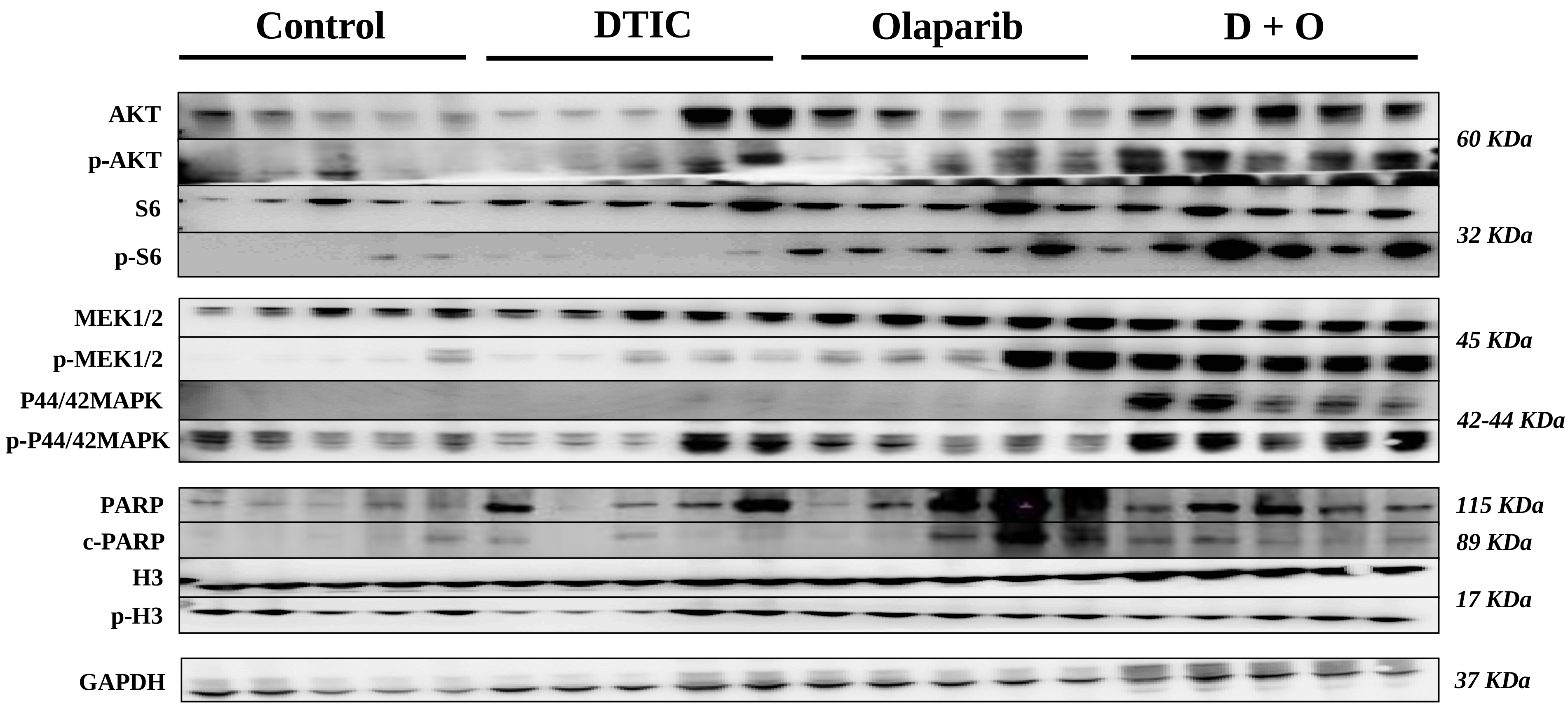

Figure S15: MM33 PDX

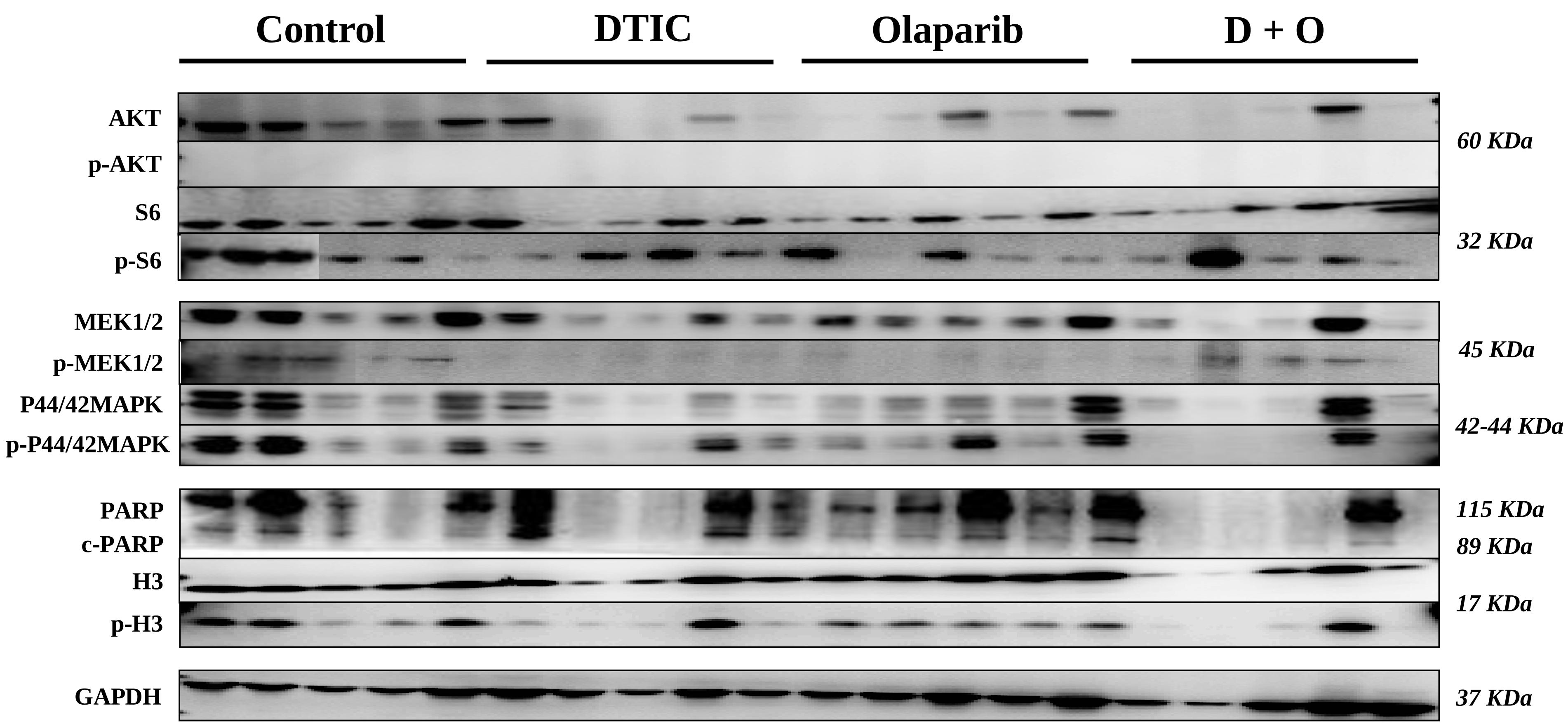

Figure S16: MM52 PDX

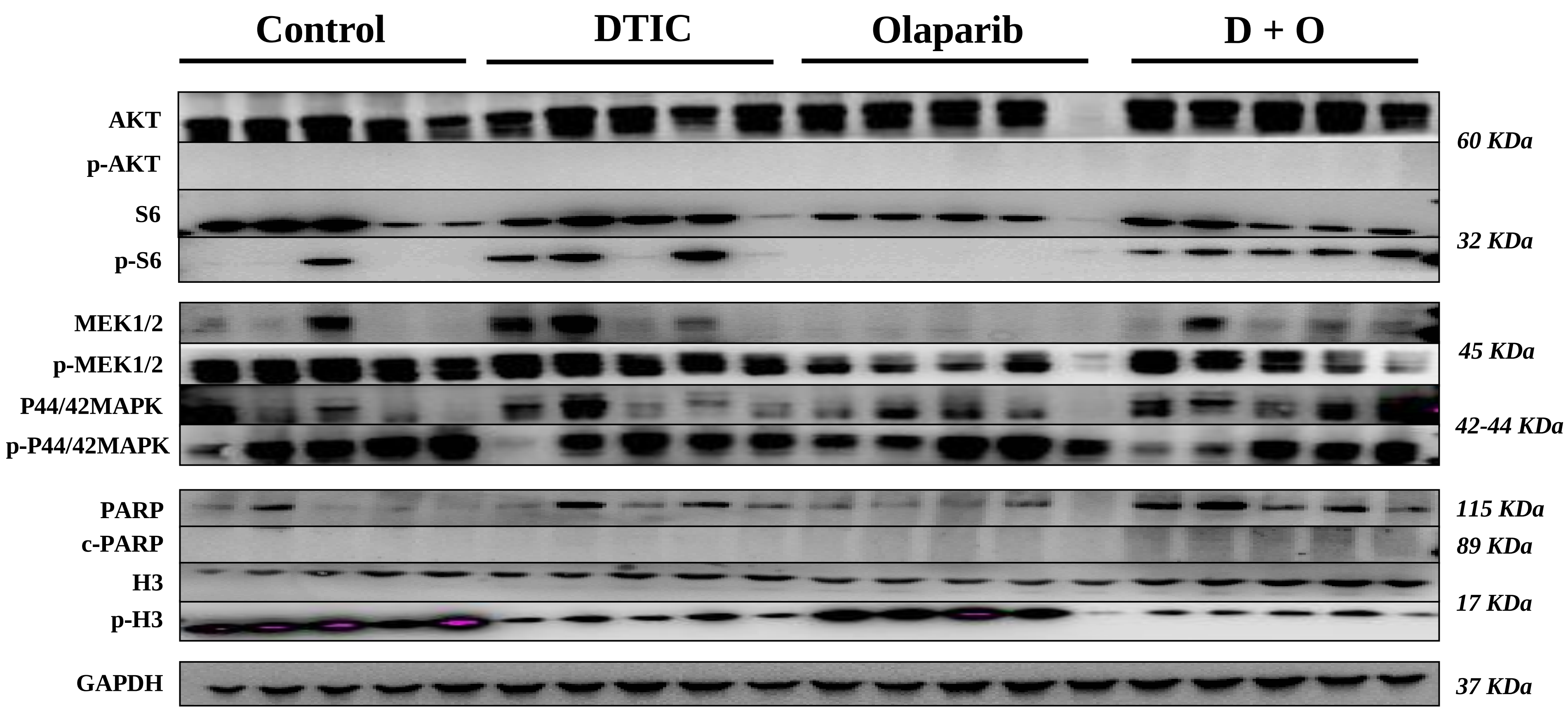

Figure S17: WB-based PARP and c-PARP protein expression

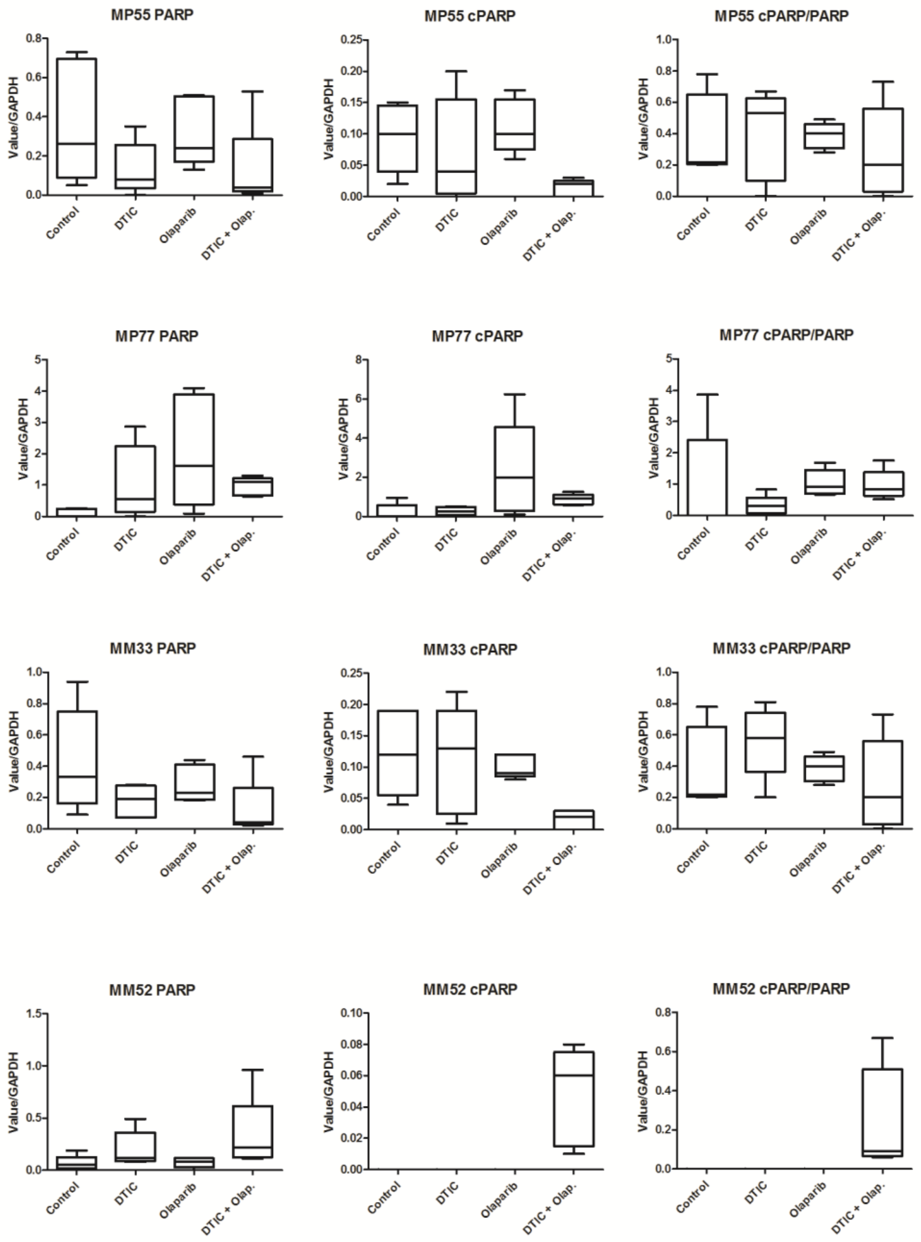

Figure S18: IHC-based apoptosis- and cell proliferation-related protein expression

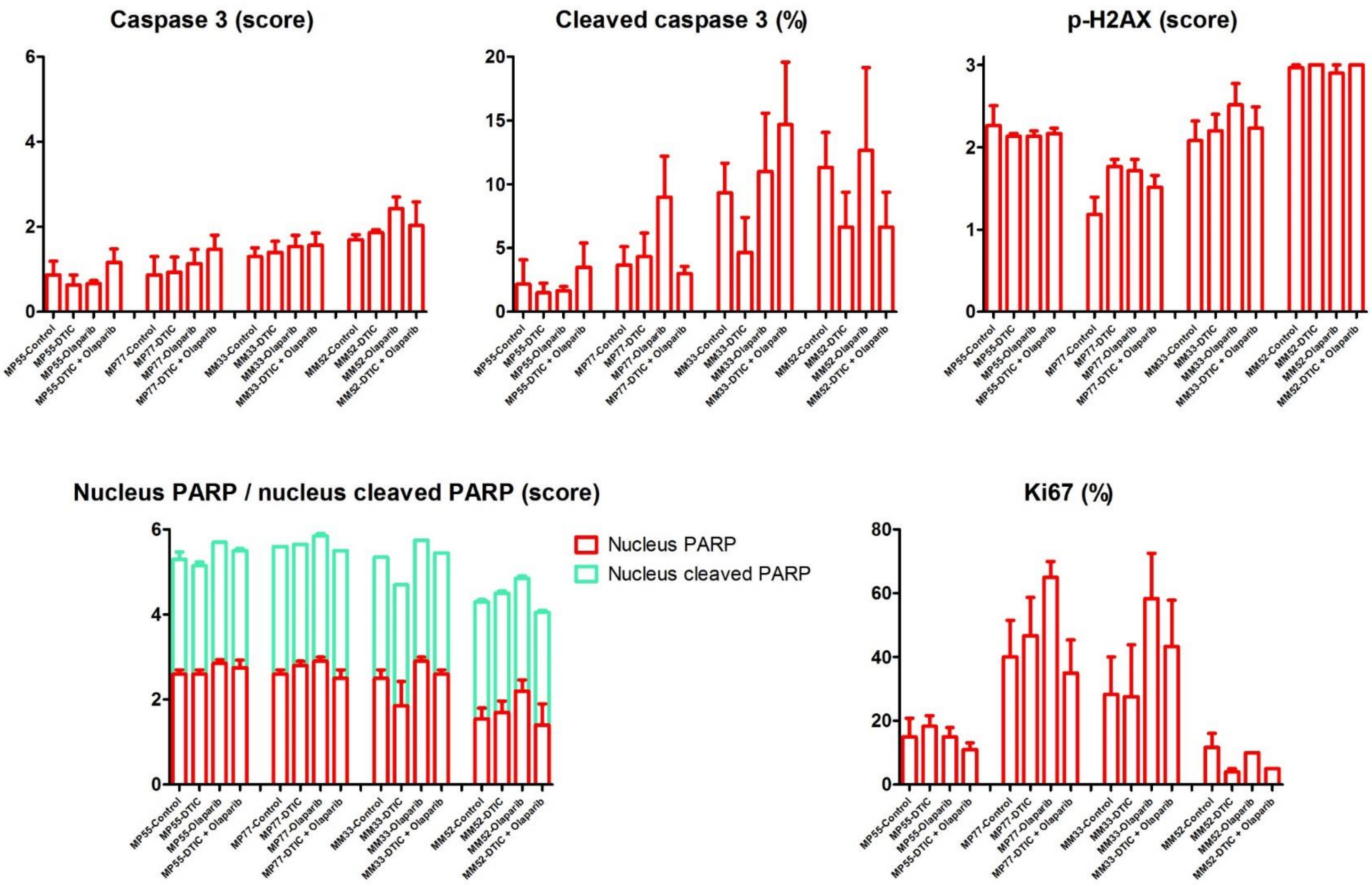

Figure S19: RPPA-based MAPK-related protein expression

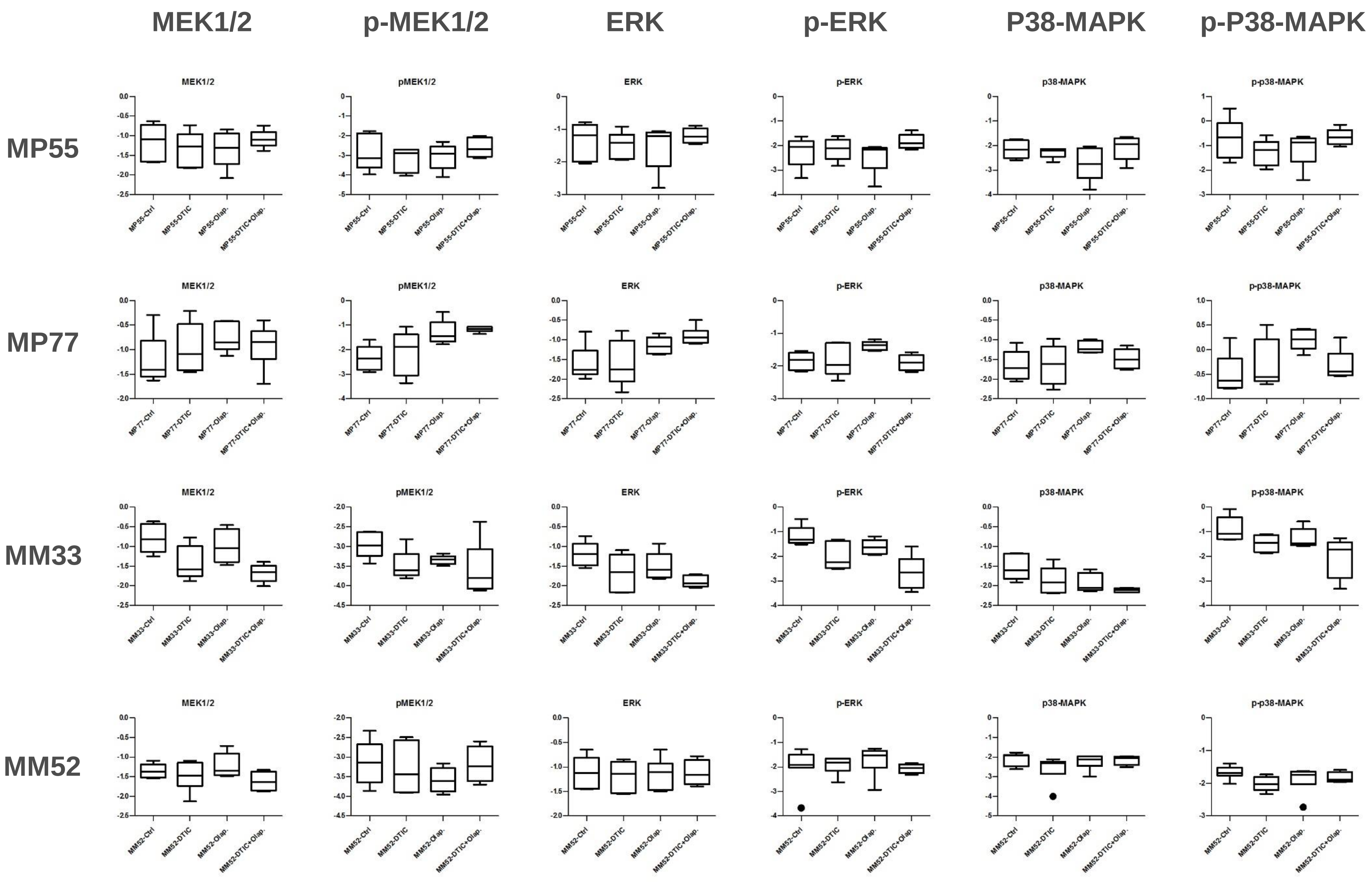

Figure S20: RPPA-based Pi3K-related protein expression

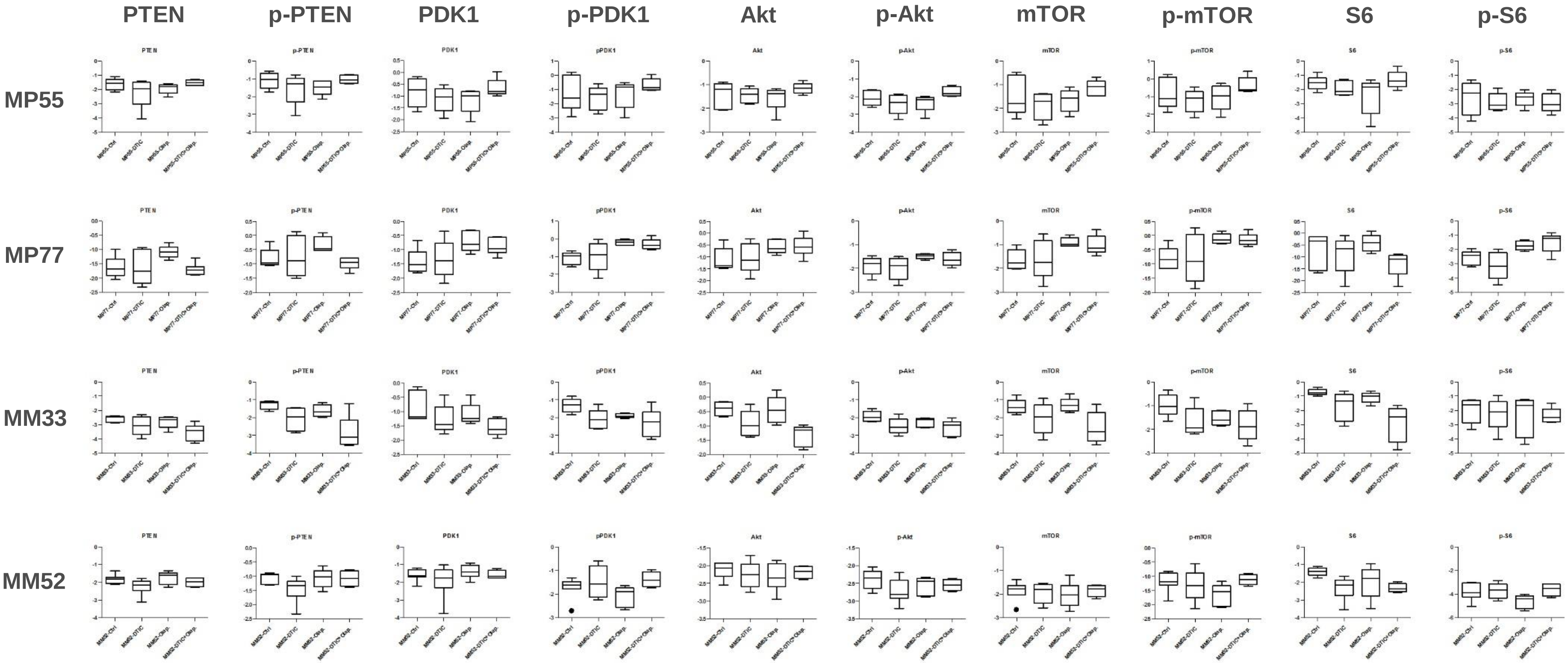

Figure S21: WB-based MAPK-related protein expression

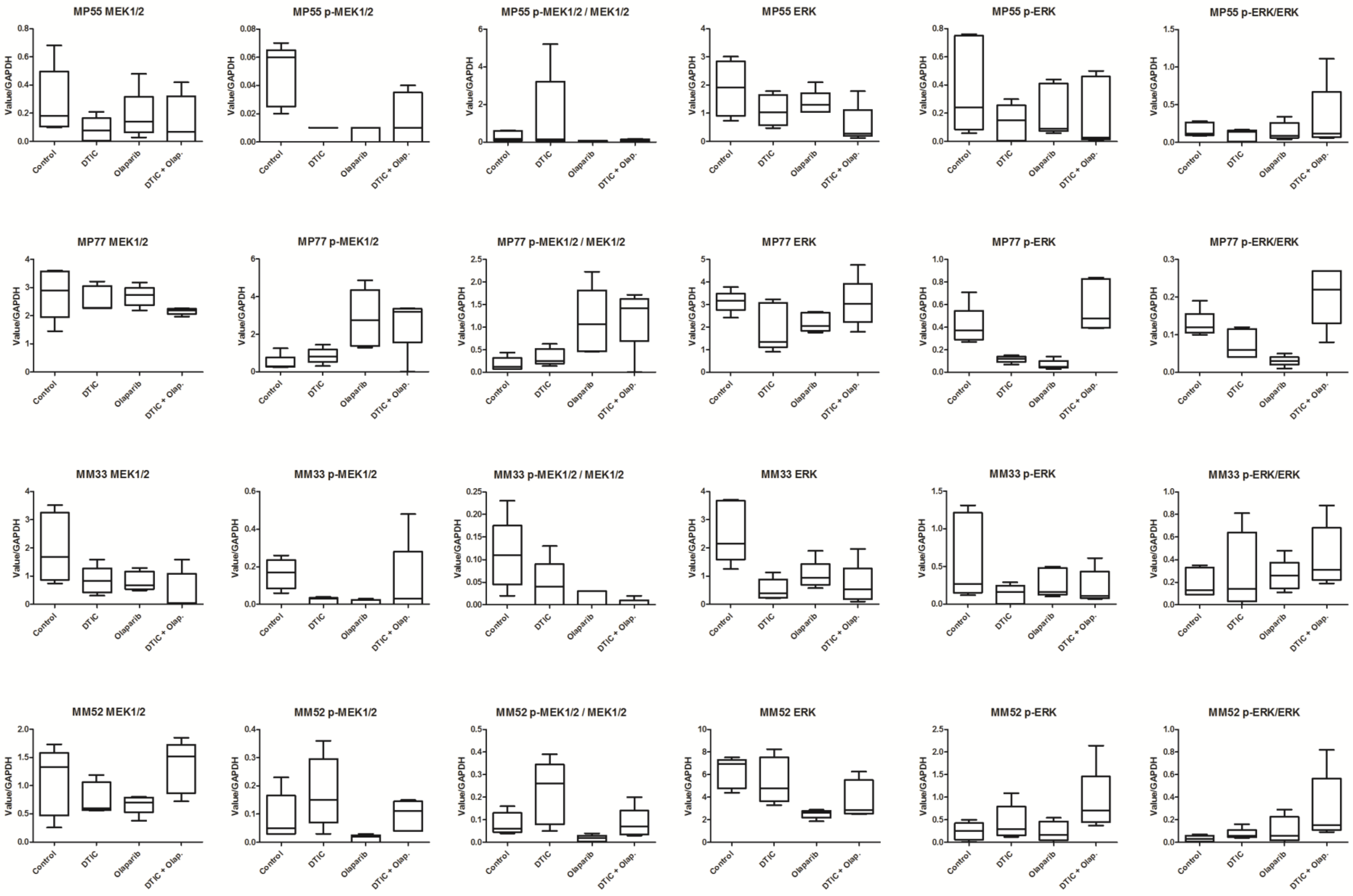

Figure S22: WB-based Pi3K-related protein expression

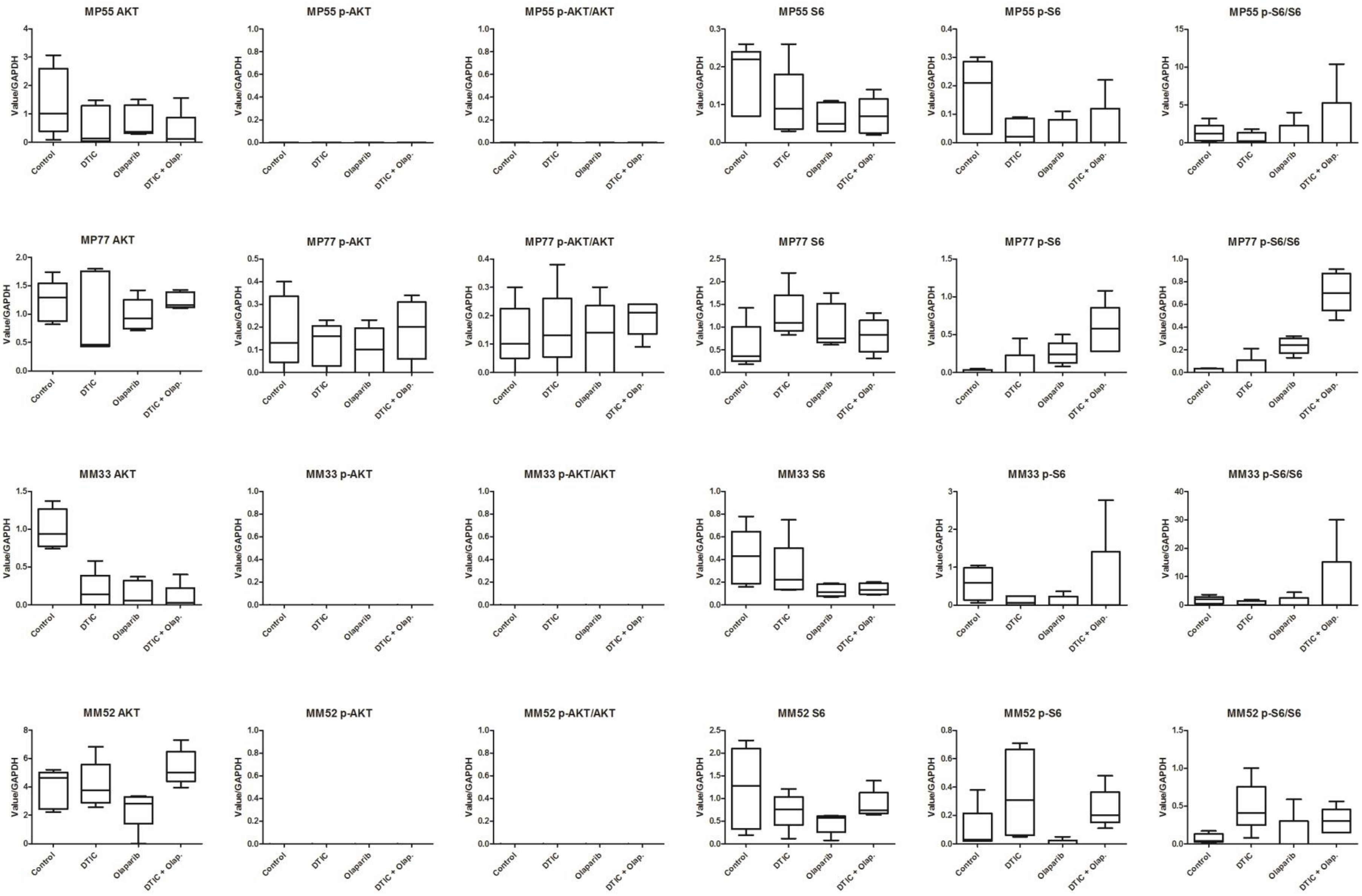

Figure S23: IHC-based MAPK-related protein expression

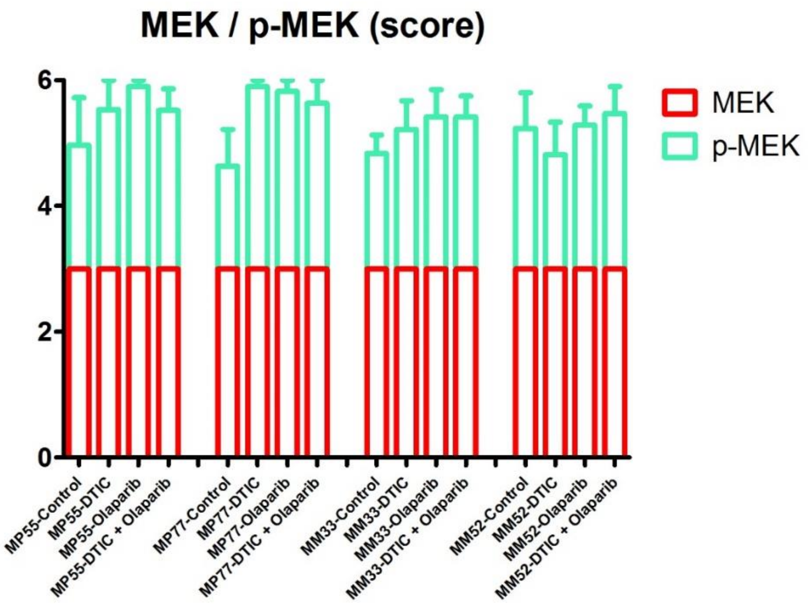

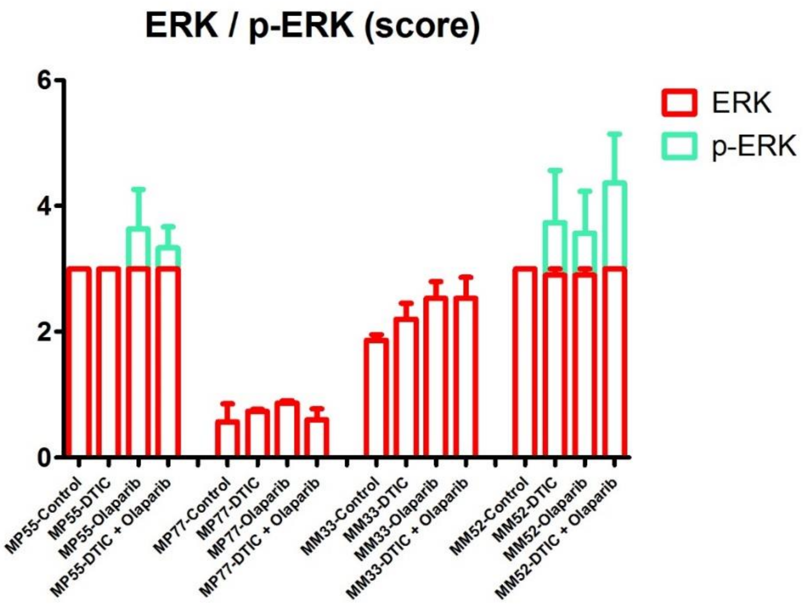

Figure S24: IHC-based Pi3K-related protein expression

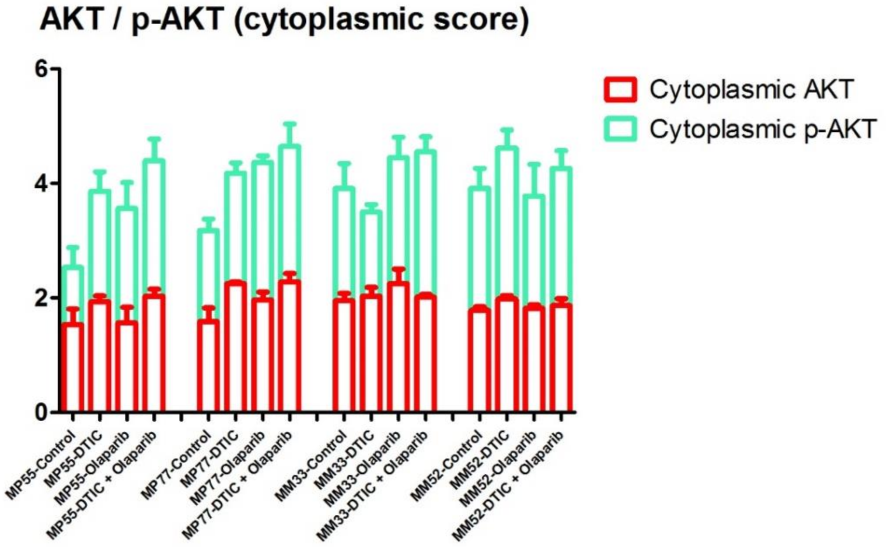

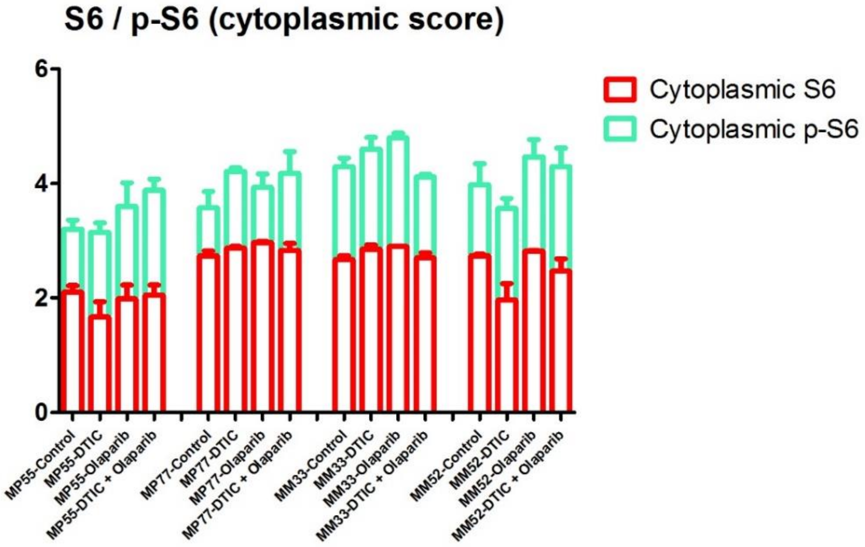

Figure S25: RPPA-based Hippo-related protein expression

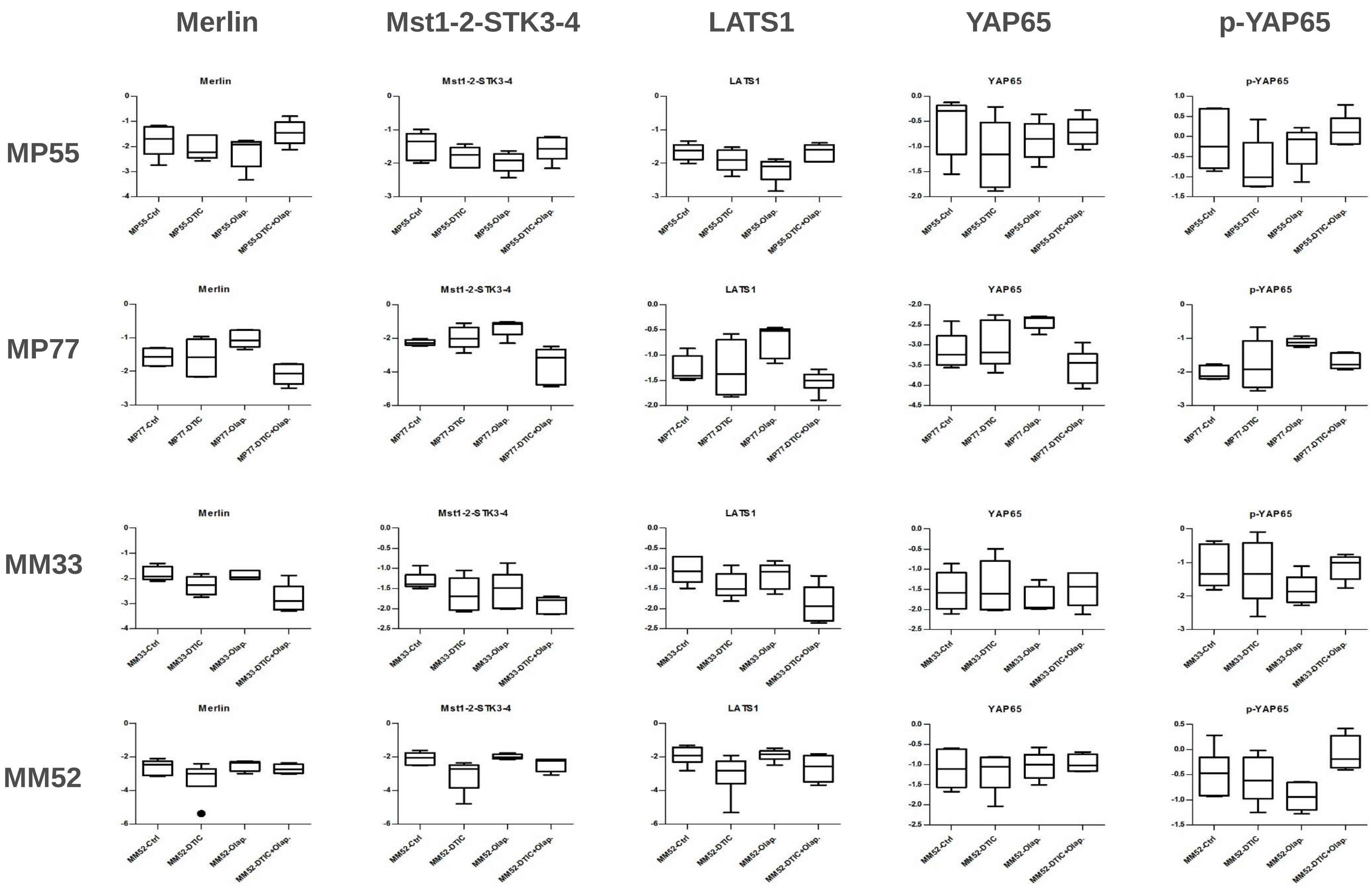

Figure S26: Hippo-related expression modifications in four treated PDXs

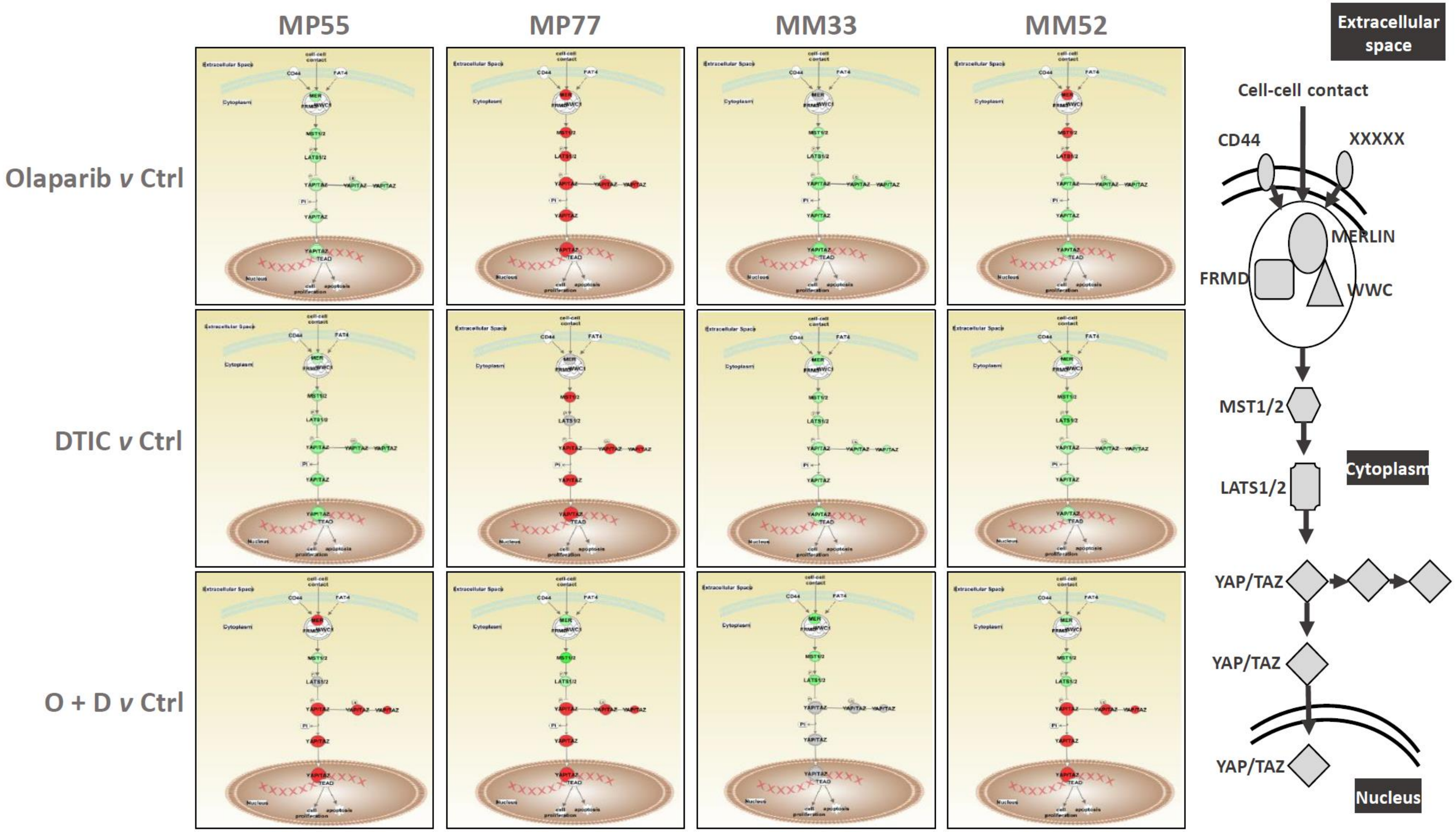

Figure S27: IHC-based Hippo-related protein expression

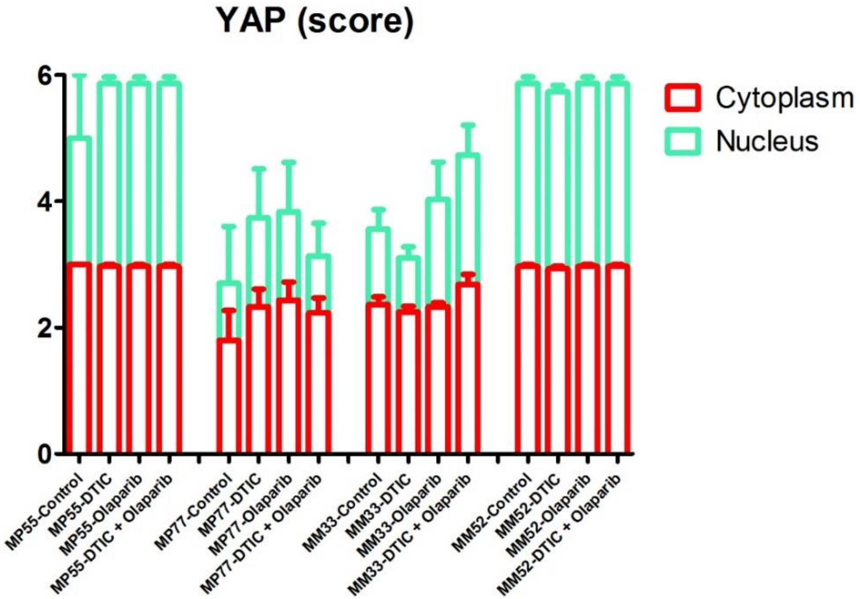

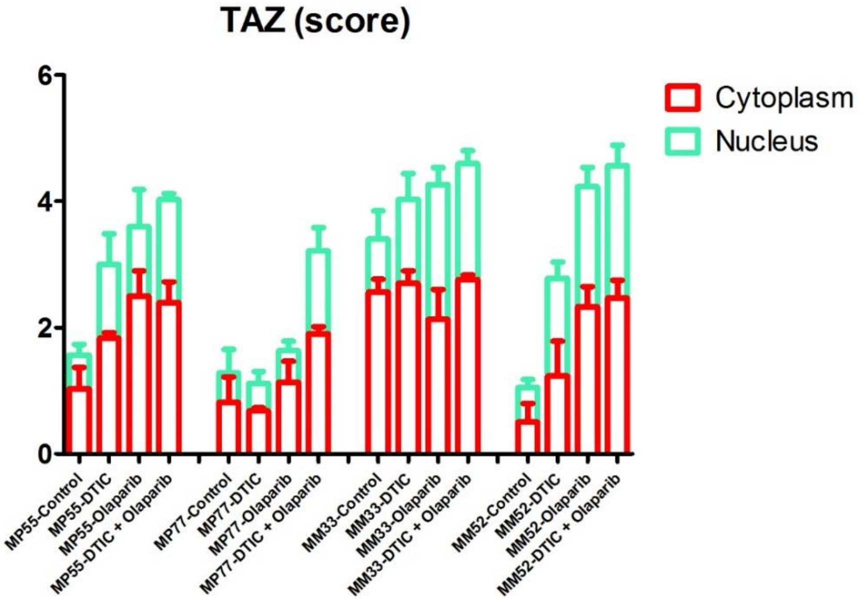

Legends to the supplementary figures

Figure S1: PARP protein expression in UM patients (P0) and their corresponding PDXs at various *in vivo* passages (P1, P4, and P9) as measured by RPPA. Protein expression was shown per model.

Figure S2: Affymetrix-based PARP family gene expression. For each transcript, box plots represent, from left to right, patient samples (P0), PDXs at passages 1, 4, and 9, and negative and positive controls.

Figure S3: Combination of olaparib and fotemustine. A-E. Growth curves (A) and ORR (B-E) of the four experimental groups, i.e. control (black), olaparib (blue), fotemustine (red), and olaparib + fotemustine (pink), respectively. F-G. Probability of progression of the four experimental groups: doubling time (F) and quadrupling time (G).

Figure S4: Combination of olaparib and everolimus. A-G. Growth curves (A-C) and ORR (D-G) of the four experimental groups, i.e. control (black), olaparib (blue), everolimus (green), and olaparib + everolimus (grey), respectively.

Figure S5: Combination of olaparib and AEB071. A-E. Growth curves (A) and ORR (B-E) of the four experimental groups, i.e. control (black), olaparib (blue), AEB071 (orange), and olaparib + AEB071 (brown), respectively. F-G. Probability of progression of the four experimental groups: doubling time (F) and quadrupling time (G).

Figure S6: Combination of olaparib and CGM097. A-E. Growth curves (A) and ORR (B-E) of the four experimental groups, i.e. control (black), olaparib (blue), CGM097 (pink), and olaparib +

CGM097 (purple), respectively. F-G. Probability of progression of the four experimental groups: doubling time (F) and quadrupling time (G).

Figure S7: Combination of olaparib and AZD0156. A-E. Growth curves (A) and ORR (B-E)of the four experimental groups, i.e. control (bleck), olaparib (blue), AZD0156 (green), and olaparib + AZD0156 (dark red), respectively. F-G. Probability of progression of the four experimental groups: doubling time (F) and quadrupling time (G).

Figure S8: Combination of olaparib and AZD6738. A-E. Growth curves (A) and ORR (B-E)of the four experimental groups, i.e. control (black), olaparib (blue), AZD6738 (orange), and olaparib + AZD6738 (green), respectively. F-G. Probability of progression of the four experimental groups: doubling time (F) and quadrupling time (G).

Figure S9: Venn diagram of the proteins significantly ($p \leq 0.05$) correlated to response to DTIC alone (blue) (MP55 + MP77 + MM52 *versus* MM33), olaparib alone (green) (MM33 *versus* MP55 + MP77 M MM52), or the DTIC+ olaparib combination (red) (MP55 + MP77 + MM33 *versus* MM52).

Figure S10: Box plots of significant protein expression between PDXs with additive efficacy (MP55-MP77-MM33) and PDX without additive efficacy (MM52) of the DTIC + olaparib combination.

Figure S11: Quantification of PARP and cleaved PARP protein expression by RPPA.

Figure S12: Quantification of apoptosis-related protein (Bcl-2, Bcl-X_L, Mcl1, Bax, and Bak) expression by RPPA.

Figure S13: Western Blots of the MP55 UM PDX treated with dacarbazine +/- olaparib.

Figure S14: Western Blots of the MP77 UM PDX treated with dacarbazine +/- olaparib.

Figure S15: Western Blots of the MM33 UM PDX treated with dacarbazine +/- olaparib.

Figure S16: Western Blots of the MM52 UM PDX treated with dacarbazine +/- olaparib.

Figure S17: Quantification of Western Blot intensities of PARP and cleaved PARP protein expression.

Figure S18: Quantification of IHC staining forapoptosis- (PARP, cleaved PARP, caspase-3, and cleaved caspase-3) and cell proliferation- (PH2AX and Ki67) related protein expression.

Figure S19: Quantification of MAPK-related protein (MEK1/2, p-MAK1/2, ERK, p-ERK, p38 MAPK, and p-p38 MAPK) expression by RPPA.

Figure S20: Quantification of Pi3K-related protein (PTEN, p-PTEN, PDK1, p-PDK1, AKT, p-AKT, mTOR, p-mTOR, S6, and p-S6) expression by RPPA.

Figure S21: Quantification of Western Blot intensities of MAPK-related protein (MEK1/2, p-MAK1/2, ERK, and p-ERK) expression.

Figure S22: Quantification of Western Blot intensities of Pi3K-related protein (AKT, p-AKT, S6, and p-S6) expression.

Figure S23: Quantification of IHC staining for MAPK-related protein (MEK1/2, p-MAK1/2, ERK, and p-ERK) expression.

Figure S24: Quantification of IHC staining forPi3K-related protein (AKT, p-AKT, S6, and p-S6) expression.

Figure S25: Quantification of Hippo-related protein (Merlin, Mst1-2-STK3-4, LATS1, YAP65, and p-YAP65) expression by RPPA.

Figure S26: Schematic representation of Hippo-related protein expression modifications in the four treated PDXs: Olaparib *versus* control, DTIC *versus* control, and Olaparib + DTIC *versus* DTIC. The intensity of color reflects fold change. Red color: higher (phospho-)protein expression in treated than in control samples. Green color: lower (phospho-)protein expression in treated than in control samples.

Figure S27: Quantification of IHC staining for YAP and TAZ protein expression.

Table S1: p values and Fold Changes (FC) in all pair comparisons of RPPA-based PARP protein expression:

Proteins	P0-P1		P0-P4		P0-P9		P1-P4		P1-P9		P4-P9	
	p	FC	p	FC	p	FC	p	FC	p	FC	p	FC
**P116.u.PARP**	0.323	-1.04	0.274	-1.13	0.480	-1.53	0.934	-1.09	0.893	-1.47	0.157	-1.35
**P25.c.PARP**	0.329	-1.65	0.914	-1.66	0.305	-3.92	0.417	-1.01	0.478	-2.37	0.579	-2.36
**P89.c.PARP**	0.150	-1.27	0.186	-1.55	0.277	-1.73	0.557	-1.22	0.917	-1.36	0.499	-1.12
**Ratio u/c.25**	0.888	-1.59	0.419	-1.47	0.570	-2.57	0.527	1.08	0.424	-1.62	0.904	-1.75
**Ratio u/c.89**	0.735	-1.22	0.988	-1.37	0.097	-1.14	0.501	-1.12	0.832	1.07	0.772	1.20

Abbreviations: P116.u.PARP, P116 uncleaved PARP; P25.c.PARP, P25 cleaved PARP; P89.c.PARP, P89 cleaved PARP; Ratio u/c.25, ratio P116 uncleaved PARP/P25 cleaved PARP; Ratio u/c.89, ratio P116 uncleaved PARP/P89 cleaved PARP.

Table S2: Treatments received by the UM PDX panel

PDXs	DTIC	Fotemustine	AEB071	AZD0156	AZD6738	CGM097	Everolimus	Olaparib
MP34				X	X	X		X
MP41	X	X						X
MP42			X					X
MP55	X			X	X	X		X
MP77	X						X	X
MM26				X	X	X		X
MM33	X		X					X
MM52	X		X					X
MM66	X	X						X
MM224				X	X	X		X
MM252				X	X	X		X

Table S3: Comparisons of RPPA-based DNA repair-related protein expression between *in vivo* experimental groups

PDXs	TGI (%)	Comparisons	NBS1	MRE11	RAD50	H2AX	p-H2AX	H3	ATM	p-ATM	p-DNA-PK	Ku80	53BP1	p-53BP1	FANCD2	p-FANCD2	Ape1	RAD51	p-Chk1	p-Chk2	P53	p-P53	SUV 39H1	MSH2	Hsp90 α	ERCC1
MP55	32	C *vs* O	NS	NS	NS	NS	NS	NS	NS	NS	NS	NS	NS	NS	NS	NS	NS	NS	NS	NS	NS	NS	NS	NS	0.04	NS
	35	C *vs* D	NS	NS	NS	NS	NS	NS	NS	NS	NS	NS	NS	NS	NS	NS	NS	NS	NS	NS	NS	NS	NS	NS	NS	NS
	65	C *vs* O + D	NS	NS	NS	0.03	NS	NS	NS	NS	NS	NS	NS	NS	NS	NS	NS	NS	NS	NS	NS	NS	NS	NS	NS	NS
	/	O *vs* O + D	NS	0.009	NS	0.003	0.03	NS	NS	NS	NS	NS	NS	NS	0.05	0.02	0.03	0.03	NS	0.03	NS	0.002	NS	NS	0.02	0.05
	/	D *vs* O + D	NS	NS	NS	NS	NS	NS	NS	NS	NS	NS	NS	NS	NS	NS	0.006	0.03	NS	NS	NS	NS	NS	NS	NS	NS
MP77	19	C *vs* O	NS	NS	NS	NS	NS	NS	0.01	0.03	NS	NS	NS	0.0005	0.03	0.04	NS	NS	NS	0.009	0.03	0.03	NS	NS	NS	NS
	73	C *vs* D	NS	NS	NS	NS	NS	NS	NS	NS	NS	NS	NS	NS	NS	NS	NS	NS	NS	NS	NS	NS	NS	NS	NS	NS
	96	C *vs* O + D	NS	NS	NS	NS	NS	0.03	NS	NS	NS	NS	NS	NS	NS	NS	NS	NS	NS	NS	NS	NS	0.04	0.02	NS	NS
	/	O *vs* O + D	0.01	0.001	NS	NS	NS	0.01	0.001	0.003	0.02	0.01	0.003	0.00007	0.008	0.01	NS	NS	0.01	0.0007	0.005	0.005	0.0009	0.0004	0.002	0.0003
	/	D *vs* O + D	NS	NS	NS	NS	NS	NS	NS	NS	NS	NS	NS	NS	NS	NS	NS	NS	NS	NS	NS	NS	NS	NS	NS	NS
MM33	36	C *vs* O	NS	NS	NS	NS	NS	NS	NS	NS	NS	NS	NS	NS	NS	NS	NS	NS	NS	NS	NS	NS	NS	NS	NS	NS
	17	C *vs* D	NS	NS	NS	NS	0.03	NS	NS	NS	NS	NS	NS	NS	NS	NS	NS	NS	NS	NS	NS	NS	NS	NS	NS	NS
	55	C *vs* O + D	0.03	NS	0.01	0.03	0.008	0.02	NS	NS	0.05	NS	0.01	0.007	0.01	0.02	0.01	NS	0.03	NS	0.001	0.04	NS	0.03	NS	0.02
	/	O *vs* O + D	NS	NS	NS	NS	NS	NS	NS	NS	NS	NS	NS	0.03	0.007	NS	0.04	NS	0.04	NS	0.008	NS	NS	NS	NS	NS
	/	D *vs* O + D	NS	NS	NS	NS	NS	0.04	NS	NS	NS	NS	NS	NS	NS	NS	NS	NS	NS	NS	NS	NS	NS	NS	NS	NS
MM52	0	C *vs* O	NS	NS	NS	NS	NS	NS	NS	NS	NS	NS	NS	NS	NS	NS	NS	NS	NS	NS	NS	NS	NS	NS	NS	NS
	51	C *vs* D	NS	NS	NS	NS	NS	NS	NS	NS	NS	0.03	NS	NS	NS	NS	NS	0.01	NS	NS	NS	NS	0.005	0.01	NS	NS
	44	C *vs* O + D	NS	0.04	NS	NS	NS	NS	NS	NS	NS	0.04	NS	NS	NS	NS	NS	NS	NS	NS	NS	NS	NS	0.002	NS	NS
	/	O *vs* O + D	NS	0.01	NS	NS	NS	NS	NS	NS	NS	NS	0.05	NS	NS	NS	NS	NS	NS	NS	NS	NS	NS	0.002	NS	0.03
	/	D *vs* O + D	NS	NS	NS	NS	NS	NS	NS	NS	NS	NS	NS	NS	NS	NS	NS	NS	NS	NS	NS	NS	NS	NS	NS	NS

Abbreviations: TGI, Tumor Growth Inhibition; C, Control group; O, Olaparib group; D, DTIC group; O + D, Olaparib + DTIC group; NS, not significant.

Significant differences of protein expression between studied groups are indicated in red.

Table S4: Comparisons of RPPA-based PARP protein expression between *in vivo* experimental groups

PDXs	TGI (%)	Comparisons	PARP	Cleaved-PARP
MP55	32	C *vs* O	NS	NS
	35	C *vs* D	NS	NS
	65	C *vs* O + D	NS	NS
	/	O *vs* O + D	0.05	NS
	/	D *vs* O + D	NS	NS
MP77 (1)	19	C *vs* O	0.003	NS
	73	C *vs* D	NS	NS
	96	C *vs* O + D	NS	NS
	/	O *vs* O + D	0.0001	0.03
	/	D *vs* O + D	NS	NS
MM33	36	C *vs* O	NS	NS
	17	C *vs* D	NS	NS
	55	C *vs* O + D	0.01	NS
	/	O *vs* O + D	0.04	NS
	/	D *vs* O + D	NS	0.03
MM52	0	C *vs* O	NS	NS
	51	C *vs* D	NS	0.04
	44	C *vs* O + D	NS	0.02
	/	O *vs* O + D	NS	0.04
	/	D *vs* O + D	NS	NS

Abbreviations: TGI, Tumor Growth Inhibition; C, Control group; O, Olaparib group; D, DTIC group; O + D, Olaparib + DTIC group; NS, not significant.
Significant differences of protein expression between studied groups are indicated in red.

Table S5: Comparisons of RPPA-based Apoptose-related protein expression between *in vivo* experimental groups

PDXs	TGI (%)	Comparisons	Bcl2	Bcl-X_L	Mcl1	Bax	Bak
MP55	32	C *vs* O	NS	NS	NS	NS	NS
	35	C *vs* D	NS	NS	NS	NS	NS
	65	C *vs* O + D	NS	NS	NS	0.04	NS
	/	O *vs* O + D	NS	0.03	NS	0.04	NS
	/	D *vs* O + D	0.04	0.03	NS	NS	0.03
MP77 (1)	19	C *vs* O	NS	0.007	0.05	NS	NS
	73	C *vs* D	NS	NS	NS	NS	NS
	96	C *vs* O + D	NS	NS	NS	NS	NS
	/	O *vs* O + D	NS	0.02	0.004	0.007	0.04
	/	D *vs* O + D	NS	NS	NS	NS	NS
MM33	36	C *vs* O	NS	NS	NS	NS	NS
	17	C *vs* D	NS	NS	NS	NS	NS
	55	C *vs* O + D	0.02	0.002	0.004	0.009	0.03
	/	O *vs* O + D	NS	NS	NS	0.003	0.03
	/	D *vs* O + D	NS	NS	NS	NS	0.007
MM52	0	C *vs* O	NS	NS	NS	NS	NS
	51	C *vs* D	NS	NS	0.03	NS	0.05
	44	C *vs* O + D	NS	NS	NS	NS	0.02
	/	O *vs* O + D	NS	NS	NS	NS	0.03
	/	D *vs* O + D	NS	NS	NS	NS	NS

Abbreviations: TGI, Tumor Growth Inhibition; C, Control group; O, Olaparib group; D, DTIC group; O + D, Olaparib + DTIC group; NS, not significant.

Significant differences of protein expression between studied groups are indicated in red.

Table S6: Comparisons of WB-based protein expression between *in vivo* experimental groups

Proteins	PDXs	O *v* C	D *v* C	O + D *v* C	O + D *v* O	O + D *v* D
**PARP**	MP55	NS	NS	NS	NS	NS
	MP77	**0.04**	NS	**0.01**	NS	NS
	MM33	NS	NS	NS	NS	NS
	MM52	NS	NS	**0.03**	**0.03**	NS
**c-PARP**	MP55	NS	NS	**0.03**	**0.01**	NS
	MP77	NS	NS	NS	NS	**0.008**
	MM33	NS	NS	**0.01**	**0.01**	NS
	MM52	NS	NS	NS	NS	NS
**H3**	MP55	**0.02**	NS	NS	NS	NS
	MP77	NS	NS	NS	NS	NS
	MM33	NS	NS	**0.008**	**0.008**	NS
	MM52	**0.04**	NS	NS	**0.04**	NS
**p-H3**	MP55	NS	NS	NS	NS	NS
	MP77	NS	NS	**0.02**	NS	NS
	MM33	NS	NS	**0.04**	NS	NS
	MM52	**0.02**	**0.008**	**0.008**	**0.03**	**0.02**
**MEK1/2**	MP55	NS	NS	NS	NS	NS
	MP77	NS	NS	NS	**0.05**	**0.02**
	MM33	NS	NS	**0.04**	NS	NS
	MM52	NS	NS	NS	**0.03**	NS
**p-MEK1/2**	MP55	Too low protein expression levels for relevant statistical analyses				
	MP77					
	MM33					
	MM52					
**ERK**	MP55	NS	NS	**0.04**	NS	NS
	MP77	**0.03**	NS	NS	NS	NS
	MM33	**0.02**	**0.008**	**0.03**	NS	NS
	MM52	**0.008**	NS	NS	NS	NS
**p-ERK**	MP55	NS	NS	NS	NS	NS
	MP77	**0.02**	**0.008**	NS	**0.02**	**0.008**
	MM33	NS	NS	NS	NS	NS
	MM52	NS	NS	**0.02**	NS	NS
**AKT**	MP55	NS	NS	NS	NS	NS
	MP77	NS	NS	NS	NS	NS
	MM33	**0.02**	**0.008**	**0.02**	NS	NS
	MM52	NS	NS	NS	**0.008**	NS
**p-AKT**	MP55	NS	NS	NS	NS	NS
	MP77	NS	NS	NS	NS	NS
	MM33	NS	NS	NS	NS	NS
	MM52	NS	NS	NS	NS	NS
**S6**	MP55	NS	NS	NS	NS	NS
	MP77	NS	NS	NS	NS	NS
	MM33	**0.03**	NS	**0.03**	NS	NS
	MM52	NS	NS	NS	**0.008**	NS
**p-S6**	MP55	NS	NS	NS	NS	NS
	MP77	**0.01**	NS	**0.01**	**0.04**	**0.03**
	MM33	NS	NS	NS	NS	NS
	MM52	NS	NS	NS	**0.01**	NS

Abbreviations: C, Control group; O, Olaparib group; D, DTIC group; O + D, Olaparib + DTIC group; NS, not significant.

Significant differences of protein expression between studied groups are indicated in red and bold.

Table S7: Comparisons of RPPA-based MAPK-related protein expression between *in vivo* experimental groups

PDXs	TGI (%)	Comparisons	MEK1/2	p-MEK1/2	ERK	p-ERK	P38-MAPK	p-p38-MAPK
MP55	32	C *vs* O	NS	NS	NS	NS	NS	NS
	35	C *vs* D	NS	NS	NS	NS	NS	NS
	65	C *vs* O + D	NS	NS	NS	NS	NS	NS
	/	O *vs* O + D	NS	NS	NS	NS	NS	NS
	/	D *vs* O + D	NS	NS	NS	NS	NS	NS
MP77 (1)	19	C *vs* O	NS	0.01	NS	0.01	0.05	0.01
	73	C *vs* D	NS	NS	NS	NS	NS	NS
	96	C *vs* O + D	NS	0.006	0.02	NS	NS	NS
	/	O *vs* O + D	NS	NS	NS	0.002	NS	NS
	/	D *vs* O + D	NS	NS	NS	NS	NS	NS
MM33	36	C *vs* O	NS	NS	NS	NS	NS	NS
	17	C *vs* D	0.04	0.05	NS	0.04	NS	NS
	55	C *vs* O + D	0.003	NS	0.005	0.005	0.02	0.03
	/	O *vs* O + D	0.02	NS	NS	0.02	NS	NS
	/	D *vs* O + D	NS	NS	NS	NS	NS	NS
MM52	0	C *vs* O	NS	NS	NS	NS	NS	0.01
	51	C *vs* D	NS	NS	NS	NS	NS	NS
	44	C *vs* O + D	NS	NS	NS	NS	NS	NS
	/	O *vs* O + D	NS	NS	NS	NS	NS	NS
	/	D *vs* O + D	NS	NS	NS	NS	NS	NS

Abbreviations: TGI, Tumor Growth Inhibition; C, Control group; O, Olaparib group; D, DTIC group; O + D, Olaparib + DTIC group; NS, not significant.
Significant differences of protein expression between studied groups are indicated in red.

Table S8: Comparisons of RPPA-based Pi3K-related protein expression between *in vivo* experimental groups

PDXs	TGI (%)	Comparisons	PTEN	p-PTEN	PDK1	p-PDK1	Akt	p-Akt°	p-Akt°°	mTOR	p-mTOR	S6	p-S6
MP55	32	C *vs* O	NS	NS	NS	NS	NS	NS	NS	NS	NS	NS	NS
	35	C *vs* D	NS	NS	NS	NS	NS	NS	NS	NS	NS	NS	NS
	65	C *vs* O + D	NS	NS	NS	NS	NS	NS	NS	NS	NS	NS	NS
	/	O *vs* O + D	NS	NS	NS	NS	NS	NS	0.05	0.04	NS	NS	NS
	/	D *vs* O + D	NS	NS	NS	NS	NS	NS	NS	NS	0.04	NS	NS
MP77 (1)	19	C *vs* O	0.03	0.04	0.02	0.003	0.02	0.008	NS	0.01	0.01	NS	NS
	73	C *vs* D	NS	NS	NS	NS	NS	NS	NS	NS	NS	NS	NS
	96	C *vs* O + D	NS	NS	NS	0.004	NS	NS	NS	0.04	0.02	NS	NS
	/	O *vs* O + D	0.001	0.003	NS	NS	0.02	NS	NS	NS	NS	0.02	NS
	/	D *vs* O + D	NS	NS	NS	NS	NS	NS	NS	NS	NS	NS	NS
MM33	36	C *vs* O	NS	NS	NS	0.03	NS	NS	NS	NS	NS	NS	0.02
	17	C *vs* D	NS	0.05	NS	0.04	NS	NS	NS	NS	NS	NS	NS
	55	C *vs* O + D	0.02	0.02	0.04	0.05	0.02	NS	0.03	0.04	NS	0.02	0.02
	/	O *vs* O + D	0.04	0.04	NS	NS	0.01	NS	NS	0.03	NS	0.03	NS
	/	D *vs* O + D	NS	NS	NS	NS	NS	NS	NS	NS	NS	NS	0.02
MM52	0	C *vs* O	NS	NS	NS	NS	0.02	NS	NS	NS	NS	NS	NS
	51	C *vs* D	NS	NS	NS	NS	NS	NS	NS	NS	NS	0.02	NS
	44	C *vs* O + D	NS	NS	NS	NS	NS	NS	NS	NS	NS	0.001	NS
	/	O *vs* O + D	NS	NS	NS	0.02	NS	NS	NS	NS	0.02	NS	0.03
	/	D *vs* O + D	NS	NS	NS	NS	NS	NS	NS	NS	NS	NS	NS

Abbreviations: TGI, Tumor Growth Inhibition; C, Control group; O, Olaparib group; D, DTIC group; O + D, Olaparib + DTIC group; NS, not significant.
° Phospho-AKT (thr 308); °° Phospho-AKT (ser 473).
Significant differences of protein expression between studied groups are indicated in red.

Table S9: Comparisons of RPPA-based Hippo-related protein expression between *in vivo* experimental groups

PDXs	TGI (%)	Comparisons	Merlin	Mst1-1-STK3-4	LATS1	YAP65	p-YAP65
MP55	32	C *vs* O	NS	NS	0.03	NS	NS
	35	C *vs* D	NS	NS	NS	NS	NS
	65	C *vs* O + D	NS	NS	NS	NS	NS
	/	O *vs* O + D	NS	NS	0.04	NS	NS
	/	D *vs* O + D	NS	NS	NS	NS	0.05
MP77 (1)	19	C *vs* O	0.01	0.02	0.02	0.02	0.0001
	73	C *vs* D	NS	NS	NS	NS	NS
	96	C *vs* O + D	0.02	0.03	NS	NS	0.04
	/	O *vs* O + D	0.0002	0.003	0.002	0.0006	0.0006
	/	D *vs* O + D	NS	0.02	NS	NS	NS
MM33	36	C *vs* O	NS	NS	NS	NS	NS
	17	C *vs* D	NS	NS	NS	NS	NS
	55	C *vs* O + D	0.01	0.003	0.01	NS	NS
	/	O *vs* O + D	0.02	NS	0.03	NS	0.03
	/	D *vs* O + D	NS	NS	NS	NS	NS
MM52	0	C *vs* O	NS	NS	NS	NS	NS
	51	C *vs* D	NS	0.04	NS	NS	0.001
	44	C *vs* O + D	NS	NS	NS	NS	0.002
	/	O *vs* O + D	NS	NS	NS	NS	0.009
	/	D *vs* O + D	NS	NS	NS	NS	NS

Abbreviations: TGI, Tumor Growth Inhibition; C, Control group; O, Olaparib group; D, DTIC group; O + D, Olaparib + DTIC group; NS, not significant.
Significant differences of protein expression between studied groups are indicated in red.

Table S10: Main characteristics of the used uveal melanoma PDXs

PDXs	Histology	Monosomy 3	+8q	*GNAQ* mutation	*GNA11* mutation	*BAP1* mutation	*SF3B1* mutation	BAP1 cell localisation	BAP1 IHC score
**MP34**	E	+	0	0	+	0	+	Nuclear	300
**MP41**	E	0	+	0	+	0	0	Nuclear	160
**MP42**	S	0	0	0	+	+	0	0	0
**MP55**	E	+	+	0	+	+	0	0	0
**MP77**	E	0	+	0	+	0	0	Nuclear	200
**MM26**	E	+	+	+	0	0	+	Nuclear	200
**MM33**	E	0	+	+	0	0	0	Nuclear	200
**MM52**	M	+	0	0	+	+	+	Cytoplasm	20
**MM66**	E	0	+	0	+	0	0	Nuclear	300
**MM224**	M	+	+	0	+	+	0	NA	NA
**MM252**	E	0	+	+	0	0	0	NA	NA

Abbreviations: NA, not available; IHC score, % of tumor cells x intensity (0 to 3).

Table S11: Compounds tested in *in vivo* experiments

Compounds	Route	Dose per administration (mg/kg)	Days of treatment
**AEB071**	PO	240	BID, 5 days/week
**AZD0156**	PO	2.5 or 5	3 days/week
**AZD6738**	PO	12.5	3 days/week
**CGM097**	PO	100	5 days/week
**Dacarbazine**	IP	40	Days 1-5, every 4 weeks
**Everolimus**	PO	5	5 days/week
**Fotemustine**	IP	20	Days 1 and 22
**Olaparib**	PO	50 or 100	5 days/week

Abbreviations: PO, per os; IP; intraperitoneal administration.

Table S12: List of the proteins studied by RPPA between patient's tumors and corresponding PDXs

ANTIBODY	SPECIES	SUPPLIER	REFERENCE
Phospho-Histone H2AX (ser139)	R	Abcam	ab2893
Histone H2AX	R	CST	2595
Mre11 (31H4)	R	CST	4847
Phospho-DNA-PK (Ser2612)	R	Epitomics	2355-1
Hsp90 alpha	R	Abcam	ab2928
Phospho-Topoisomerase II a (Thr1343)	R	Epitomics	1871-1 / ab52853
ATM	R	Epitomics	1549-1
ERCC1	R	CST	3885
Cleaved PARP (Asp214) p25	R	Epitomics	1051-1
Phospho-ATM (ser1981)	R	Novus	NB110-55475
MSH2 (D24B5)	R	CST	2017
53BP1	R	CST	4937
Rad50	R	CST	3427
MDM2 [EP16627]	R	Abcam	ab178938
Phospho-S6 Ribosomal Protein (Ser240/244)	R	CST	2215
YAP65	R	Epitomics	2060-1
Phospho-S6 Ribosomal Protein (Ser235/236)	R	CST	2211
Akt (pan) (C67E7)	R	CST	#4691
Akt	R	CST	9272
Phospho-Akt (Thr308) (D25E6)	R	CST	13038
PTEN (D4.3) XP	R	CST	9188S
phospho-mTOR (Ser2448)	R	Abcam	ab109268
Phospho-PTEN (ser380/Thr382/383)	R	CST	9554
Bcl2	R	CST	2876
Bak	R	Epitomics	1542-1
mTOR	R	Abcam	ab51089
Mst1/2 / STK3/4	R	Bethyl	A300-468A
Bcl-xL	R	Epitomics	1018-1
Mcl-1	R	Santa-Cruz	SC-819
Phospho-YAP65 (Ser127)	R	CST	4911S
PARP uncleaved p116	R	Epitomics	1077-1 / ab32378
Bax (D2E11)	R	CST	#5023
Phospho-PDK1 (Ser241)	R	CST	3061
PDK1 (D37A7)	R	CST	5662
Phospho-Akt (Ser473) (193H12)	R	CST	4058

NBS1 p95	R	CST	3002
Merlin	R	Epitomics	3357-1
Ape1	R	CST	4128
p38 MAPK	R	Epitomics	1544-1
Topoisomerase II alpha	R	Epitomics	1826-1
Phospho-53BP1 (Ser1778)	R	CST	2675
Phospho-MEK1/2 (Ser217/221)	R	CST	9154
Phospho-Estrogen Receptor alpha (Ser118)	R	Epitomics	1091-1
p53	R	CST	9282
Ezrin	R	Epitomics	2255-1
LATS1	R	Bethyl	A300-478A
MEK1/2	R	CST	9122S
Phospho-Chk2 (Thr68)	R	CST	2197
Phospho-p38 MAPK (Thr180/Tyr182)	R	CST	4631
Phospho-p44/42 MAPK (Thr202/Tyr204)	R	CST	4377 (197G2)
Estrogen receptor alpha (D8H8)	R	CST	8644
p44/42 MAPK	R	CST	9102
Phospho-Chk1 (Ser280)	R	CST	2347
Phospho-Ezrin (Thr567) / Radixin (Thr564)/Moesin (Thr558)	R	CST	3141
Phospho-FANCD2 (Ser222)	R	CST	4945
S6 Ribosomal Protein (5G10)	R	CST	2217
FANCD2	R	Epitomics	2986-1
Histone H3 trimethylated K9 (H3K9me3)	R	Upstate (Millipore)	07-442
Phospho-p53 (Ser15)	R	CST	9284
SUV39H1	R	CST	8729
Rad51 (D4B10)	R	CST	8875
Progesterone receptor	R	Epitomics	1483-1
Ku80 (C48E7)	R	CST	2180
Phospho-Progesterone Receptor (Ser190)	R	Epitomics	2258-1

Table S13: List of the proteins studied by Western Blots and their corresponding used antibodies

Signaling pathway / function	Protein	Reference of the antibody
Control	GAPDH	Cell Signaling Technology, #2118
PARP and apoptosis	PARP	Abcam, # ab32378
	Cleaved PARP	Cell Signaling Technology, #9541
	Histone H3	Cell Signaling Technology, #9717
	p-Histone H3	Abcam, # ab32107
MAPK	MEK1/2	Cell Signaling Technology, #9126
	p-MEK1/2 (ser217/221)	Cell Signaling Technology, #9154
	ERK	Cell Signaling Technology, #9102
	p-ERK (Thr202/Tyr204)	Cell Signaling Technology, #4370
Pi3K	AKT	Cell Signaling Technology, #9272
	p-AKT (ser473)	Cell Signaling Technology, #4058
	S6	Cell Signaling Technology, #2117
	p-S6 (Ser235/236)	Cell Signaling Technology, #2211

Table S14: List of the proteins studied by IHC and their corresponding used antibodies

Proteins	Origin	Clone	pH	Concentration	Time of incubation
**AKT**	Santa Cruz	sc-8312	pH6	1/100e	1 hour
**Caspase 3**	Abcam	ab4051	pH6	1/50e	1 hour
**Cleaved PARP**	Abcam	ab32064	pH6	1/100e	1 hour
**Cleaved-caspase 3**	Cell signaling	#9661	pH6	1/250e	1 hour
**ERK**	Abcam	ab32537	pH6	1/200e	1 hour
**ki67**	Dako	F7268	pH9	1/100e	1 hour
**MEK1/2**	Abcam	ab32091	pH9	1/75e	1 hour
**P-AKT**	Cell signaling	#9277	pH9	1/50e	Overnight (4°C)
**PARP**	Abcam	ab32138	pH6	1/150e	1 hour
**p-ERK**	Abcam	ab194770	pH9	1/250e	1 hour
**p-H2AX**	Cell signaling	#9718	pH9	1/100e	1 hour
**p-MEK1/2**	Abcam	ab96379	pH9	1/600e	1 hour
**p-S6**	Cell signaling	#4858	pH6	1/100e	1 hour
**S6**	Abcam	ab40820	pH6	1/300e	1 hour
**TAZ**	Abcam	ab110239	pH6	1/350e	1 hour
**YAP**	Protein tech	13584-1-AP	pH6	1/2000e	1 hour
